# Heterogeneous Iris One-to-One Certification with Universal Sensors Based On Quality Fuzzy Inference and Multi-Feature Fusion Lightweight Neural Network

**DOI:** 10.3390/s20061785

**Published:** 2020-03-23

**Authors:** Liu Shuai, Liu Yuanning, Zhu Xiaodong, Huo Guang, Wu Zukang, Li Xinlong, Wang Chaoqun, Cui Jingwei

**Affiliations:** 1 College of Computer Science and Technology, Jilin University, Changchun 130012, China; lxs940518@163.com (L.S.); lyn@jlu.edu.cn (L.Y.); wuzk2115_jlu@163.com (W.Z.); 2 Key Laboratory of Symbolic Computation and Knowledge Engineering of Ministry of Education, Jilin University, Changchun 130012, China; xinlong19@mails.jlu.edu.cn (L.X.); wangcq19@mails.jlu.edu.cn (W.C.); neko_185490533@163.com (C.J.); 3 College of Computer Science, Northeast Electric Power University, Jilin 132012, China; yanhuo1860@126.com; 4 College of Software, Jilin University, Changchun 130012, China

**Keywords:** heterogeneous iris one-to-one certification, universal sensors, quality fuzzy inference, iris quality knowledge concept construction mechanism, lightweight neural network, multi-source feature fusion mechanism, feedback learning mechanism

## Abstract

Due to the unsteady morphology of heterogeneous irises generated by a variety of different devices and environments, the traditional processing methods of statistical learning or cognitive learning for a single iris source are not effective. Traditional iris recognition divides the whole process into several statistically guided steps, which cannot solve the problem of correlation between various links. The existing iris data set size and situational classification constraints make it difficult to meet the requirements of learning methods under a single deep learning framework. Therefore, aiming at a one-to-one iris certification scenario, this paper proposes a heterogeneous iris one-to-one certification method with universal sensors based on quality fuzzy inference and a multi-feature entropy fusion lightweight neural network. The method is divided into an evaluation module and a certification module. The evaluation module can be used by different devices to design a quality fuzzy concept inference system and an iris quality knowledge concept construction mechanism, transform human logical cognition concepts into digital concepts, and select appropriate concepts to determine iris quality according to different iris quality requirements and get a recognizable iris. The certification module is a lightweight neural network based on statistical learning ideas and a multi-source feature fusion mechanism. The information entropy of the iris feature label was used to set the iris entropy feature category label and design certification module functions according to the category label to obtain the certification module result. As the requirements for the number and quality of irises changes, the category labels in the certification module function were dynamically adjusted using a feedback learning mechanism. This paper uses iris data collected from three different sensors in the JLU (Jilin University) iris library. The experimental results prove that for the lightweight multi-state irises, the abovementioned problems are ameliorated to a certain extent by this method.

## 1. Introduction

This paper takes lightweight one-to-one certification of multi-state iris in the same environment as the research object, and proposes a one-to-one certification method with universal sensors for heterogeneous irises based on quality fuzzy inference and multi-feature entropy fusion lightweight neural networks. The feedback learning mechanism enables the overall process to dynamically adjust the concept of the iris in the fuzzy system and the category labels in the recognition function as the number and quality of irises change. 

The prerequisites for this method are listed as follows:

1. The iris acquisition status and acquisition environment change, and this change cannot be predicted, which causes certain defocusing, deflection, shadowing and other problems. The dimensions of the captured images are 640 × 480;
2. The number of iris categories is lightweight (all iris libraries contain dozens of categories, and each category contains only a few thousand pictures);
3. To ensure the accuracy of lightweight certification, testers are allowed to collect multiple times, and thus, it is allowable to appropriately increase the false rejection rate;
4. The number of training irises can reach several thousand, but the types of training irises (degree of defocus, illumination, strabismus effect, etc.) are not classified; they are directly mixed, belonging to mixed data;
5. The subject tester is a living human.

**Figure 1:**
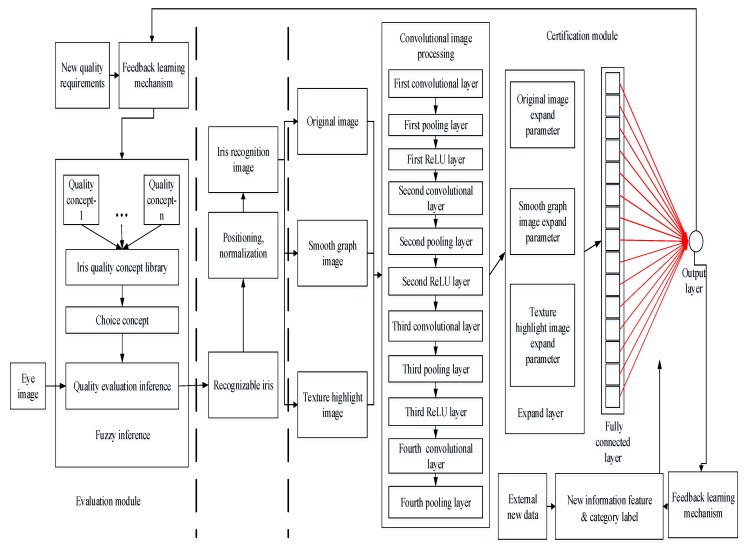
The overall working process of the method in this paper.

The overall working process of the method proposed in this paper is shown in Figure 1.

The whole process of the method in this paper is divided into an evaluation module and a certification module, and they are connected together through a transition phase of iris positioning.

1. **Evaluation module:** The evaluation module is a set of general processes that can be run on the irises collected by different acquisition instruments. This paper designs a quality concept fuzzy inference method based on a pure fuzzy logic system to select a recognizable iris. First, based on the existing training iris, a mechanism for the eye concept base and the qualified iris image concept base is proposed, and the subjective cognition of a person with normal thinking initially transforms the concept of an iris into a digital logic concept of the computer system. When evaluating the quality of the test iris, suitable concepts are selected according to the iris recognition requirements, and a quality inference machine is used to determine whether the test iris can be used for iris recognition. After the recognizable iris is processed, it is input into the recognition model. With the analysis of certification error results and the emergence of new quality requirements, a feedback learning mechanism for quality evaluation is designed for the updating and correction of knowledge. Existing conceptual labels are checked according to changes in the number of irises, errors of the original labels are corrected, and label expansion is implemented according to changes in quality requirements. The recognizable iris image determined by the evaluation module is located, normalized, and converted into a 180 × 32 dimensional iris recognition area.
2. **Certification module:** Based on the convolutional neural network structure, a neural network structure with lightweight layers is designed for iris certification. A feature representation mechanism based on multi-source features is proposed. The image of the iris certification area is processed by a smoothing algorithm and a texture highlighting algorithm. Three different iris images are formed as multi-source features. Each iris image passes through 12 layers of an image-processing network consisting of a convolutional layer, pooling layer, ReLU layer, and expansion layer. Finally, each iris image forms 15 expanded parameters and a total of 45 expanded parameters in the expansion layer. In the certification module, the expanded parameters of the three images are fused by average fusion through the feature fusion layer to form 15 recognition parameters. The certification function is designed based on the sigmoid function [1], and the statistical information is used to calculate the certification parameter information entropy. According to the information entropy, the certification function parameters are designed as the category labels. In the fully connected layer, one-to-one certification is finally performed through the certification function, and finally, the final result is output through the output layer. With the analysis of certification error results and the emergence of new iris data, in order to modify the information entropy and new category labels, a feedback learning mechanism for the certification module is designed.

Compared with the current research, the innovation and research significance of this method in terms of improving the accuracy of one-to-one certification are described as follows:

1. **The problem of the heterogeneous unsteady-state iris:** In quality evaluation, design of the iris quality knowledge concept mechanism must minimize the impact of fixed threshold decisions and use the objective iris features to set the concept. It must not require a fixed index for mechanical evaluation, but set the concept of quality based on the actual environment and identification needs for conceptual reasoning. In feature expression, a multi-source feature fusion mechanism is designed, feature fusion is performed from a multi-source perspective, and discrimination between different types of features is increased. The information entropy is used to design the recognition function category label parameters, realize the statistical recognition of the multi-state iris in the mixed data set, expand the range of recognizable irises, and improve the accuracy of unsteady heterogeneous iris certification.
2. **For the correlation between the various links in the whole process:** This paper uses the non-template matching mode as the basis for the design of the certification model. It does not focus on a specific algorithm for research, but mainly focuses on the research of the protocol mechanism, and proposes a set of solutions for the whole process of iris certification from image acquisition to result output. In constructing the knowledge base, reasoning process, certification process, and a series of agreement mechanisms were proposed to connect the links with each other and fully consider the correlation between the certification steps. Aiming at resolving the instability of the unsteady-state iris and making the scheme universal, lightweight heterogeneous certification can be realized; that is, the iris images collected under different environments by different sensors can be recognized by the scheme.
3. **Limitations on iris dataset size and situation classification:** Based on improvement of the positive certification process, this paper designs a feedback learning mechanism in the revision of the reverse overall process. Via professional analysis by human operators and data feedback from the computer system, the error certification situation and new possible external conditions are added. In this way, the process of performing a response and adjusting or correcting the knowledge base concept and the entropy features of the category labels enables the entire certification process to achieve dynamic adjustment, thus improving the forward certification and reverse correction of deep learning frameworks under the data set constraints.

The structure of this paper is as follows. Section 1—the introduction—explains the research content, prerequisites, and innovations of this paper. Section 2—background and related work —explains the relevant background of this article’s research content and domestic and foreign trends, and then explains the research value of this paper. Section 3—quality fuzzy inference —details of the process of iris quality evaluation in this paper. Section 4—heterogeneous iris one-to-one certification—introduces the process of certification in this paper in detail. Section 5—experiments and analysis—analyzes the advantages of the method in this paper in terms of the structural significance and certification effects and compares the current algorithms with various algorithms to highlight the advantages of the method under the prerequisites of this paper. Section 6— conclusion—summarizes the methods and experiments in this paper.

## 2. Background and Related Work

Iris recognition is currently one of the highest-security technologies recognized in the field [1]. In actual iris recognition, there are a variety of use scenarios, which are mainly divided into several situations:

In the iris collection state, the main settings are the collection status (collection posture, collection distance) and the external environment (illumination), which can be divided into four categories:

1. Unconstrained state in the same environment: Iris acquisition is performed based on a lack of restriction of the acquisition posture of the acquisition target person, and the external environment is not changed for iris acquisition;
2. Constrained state in the same environment: Iris acquisition is performed based on the restriction of the acquisition posture of the acquisition target person, and the external environment is not changed for iris acquisition;
3. Constrained state with environmental change: Iris acquisition is performed based on the restriction of the acquisition posture of the acquisition target person, and the external environment is changed for iris acquisition;
4. Unconstrained state with environmental change: Iris acquisition is performed based on a lack of restriction of the acquisition posture of the acquisition target person, and the external environment is changed for iris acquisition.

According to the recognition method, the process of recognition can be divided into two types:

1. Template matching: Coding the test iris with a single storage template iris to get the final conclusion;
2. Non-template matching: The features of the iris are formed into a cognitive concept, which is incorporated into neural network architectures such as deep learning [2] in the form of parameters, etc., to achieve non-coding matching recognition.

The essential purpose of use can be divided into two categories:

1. One-to-one certification: It is used to determine whether the test iris and the template iris belong to the same person;
2. One-to-many recognition: The iris of the test person is matched with multiple template irises, and the identity of the test person must be accurately identified.

There are separate process frameworks for completing iris recognition in various classification situations, and no matter what type of iris recognition is used, they all need to ensure high accuracy. The following aspects still require further breakthroughs.

**Figure 2:**
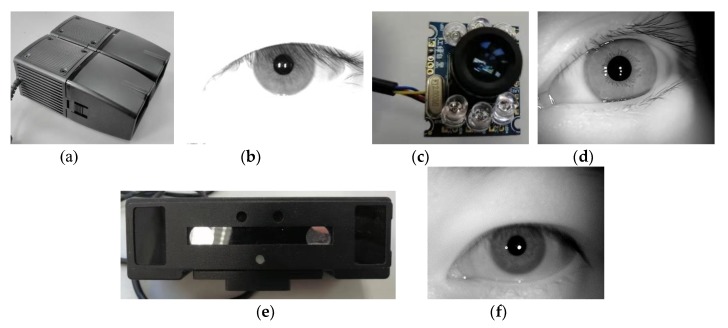
Eye acquisition sensors. (**a**) NIR acquisition sensor, (**b**) NIR acquisition sensor for ideal images, (**c**) ordinary optical acquisition sensor, (**d**) ordinary optical acquisition sensor for ideal images, (**e**) ordinary optical acquisition sensor for upgrading sensors, (**f**) ordinary optical acquisition sensor with upgraded sensors corresponds to ideal images.

1. **Research on the whole structure of iris recognition:** In the current research on iris recognition, template matching methods are basically aimed at a specific scene in image acquisition [3], quality evaluation [4], localization normalization [5], feature expression [6], and recognition [7] and take one or more of these steps as the core to improve and further improve performance. This method mechanically separates the steps, ignores the agreement between the algorithms, and limits effect on the overall performance accuracy. Non-template matching methods based on neural network structures such as deep learning explore the internal connections of certain steps, nesting them together to form a whole, and not necessarily completing all steps, thereby improving the accuracy. However, there is no qualitative and quantitative analysis method for the clear correlation between the steps in the overall structure of iris recognition. It seriously affects the demand for improving the performance of the overall recognition system.
2. **Iris multi-state and single-source feature expression:** Most training and learning recognition models in the current iris recognition algorithms are based on publicly known iris training sets or manually set iris labels. However, in the actual shooting process, multi-state heterogeneity is expected to occur between the template image and the collected image. There are many reasons for the multi-state heterogeneity, and they are divided into three main aspects:
  a. The acquisition sensor specifications are different (e.g., an NIR sensor [8] and ordinary optical sensor [9]; iris sample pictures taken by different cameras are shown in Figure 2).
  b. The collection environment is different because the iris status is unstable under different environments (illumination, etc.), which affects the relative relationship between the iris textures.
  c. The acquisition status of the iris is different. Because the acquisition status of the target person varies at different times, disturbances such as defocusing and deflection occur.

**Figure 3:**
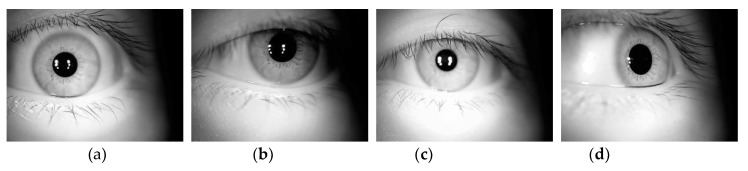
Examples of multi-state irises: (**a**) normal iris, (**b**) iris with dark condition, (**c**) iris with defocused condition, (**d**) iris with deflected condition.

Because the algorithm applied in the process of iris processing and recognition takes on a black box state, it is impossible to predict the outcome, which makes it difficult to design a unified process for a variable and unsteady iris using fixed parameters and to subsequently form selected effects for iris quality, localization, and feature expression; this difficulty leads to the unsteady nature of iris features. The example of multi-state irises (taking the same device and the same person as an example) is shown in Figure 3.

It can be observed from Figure 3 that even the same device and the same person cannot guarantee the final appearance of the iris feature, which can affect the expression of the iris feature. Therefore, research into the impact of a multi-state iris on the setting of iris labels requires further examination.

3. **Concept label setting and universal recognition of heterogeneous iris datasets:** Currently, two main forms of iris concept label setting exist. The first is statistical learning methods [10]: a reasonable solution is obtained that meets the vast majority of requirements through the analysis of a notably large amount of data in the same type of situation. The second is cognitive learning methods [11]: by imitating the process of human learning, labels are ascribed to things to form the concept of things. The statistical learning method is currently the most commonly used method, and it applies a good combination of deep learning and other aspects. However, this type of method has high requirements for data preparation. In addition to requiring large amounts of data, this type of method also requires a clear division of the type of irises, and the limitations of the existing iris dataset size and situation classification make it difficult to meet a single deep learning frame. The amount of data required for the learning method under the learning framework also makes it difficult to support the establishment of a self-improvement process from forward recognition to the reverse. In addition, the accumulation of multi-state irises cannot be completed in a short time, which greatly limits the role of statistical learning. Although cognitive learning can reduce the need for data volume, the relationship between the iris labels and unknown environments needs further research. Because the hardware configuration between different collectors and the environment in which they are collected is expected to vary at different times, and the algorithm itself has prerequisites for use, this situation makes the recognition effect of the same algorithm differ substantially in different situations, thus greatly increasing the design complexity of iris recognition algorithms.

These problems make the use of the iris more accurately in one-to-one certification. To solve these problems, certain progresses have been reported in current research on the expression and recognition of iris features.

In the current research, there have been many practically tested pattern recognition frameworks. The existing framework is improved and tested through the public iris set, which proves that the framework is feasible, such as iris specific Mask R-CNN [12] and deep learning frameworks such as capsule neural networks under lightweight data structures [13], as well as existing deep learning frameworks specifically for iris recognition, such as DeepIris [14] and DeepIrisNet [15]. In research on the multi-state iris, the unsteady-state features are transformed into steady-state features through image processing and other methods [16], and the iris features are expressed through multiple recognition methods and weighted fusion [17] or the final result is obtained based on a credibility decision [18]. To set the iris concept labels, research into biomimetic cognition is an important direction; i.e., the determination of how to first summarize a feasible recognition model in the case of a small number of initial training samples, which increases in the number of recognitions and available training samples, as well as how to further effectively judge whether the current structure cannot meet the existing situation and requires users to retrain [19]. A current proposal is to set the iris label for the statistical cognitive learning method of unclassified mixed data [20] and apply the MiCoRe-Net neural network architecture [21] to eye concept reconstruction. In addition, for the label correction of the existing neural network architecture, an error correction code-based label optimization method [22] and a feedback mechanism-based label correction method [23] have been proposed. To make up for the lack of data in the dataset through transfer learning [24], in the study of iris generality, heterogeneous iris recognition is the main research direction [25]. First, by changing the internal structure of the algorithm, the device independence of the iris image is improved [26]. Second, the correction of images (blurring, displacement, etc.) using algorithms [27] improves the feasibility of multi-type iris recognition and further improves the environmental independence. In addition, starting from the acquisition sensor, improving the acquisition status during image acquisition and improving the iris quality from the beginning is also one of the current research directions of iris generality [28].

Previous studies have achieved good results in various experiments, but selected problems remain, and thus, there is a need to improve the accuracy of iris one-to-one certification.

1. The existing structural framework is designed to process a certain type of data, and the design purpose of the framework is not necessarily aimed at iris recognition. When the existing framework is used in iris recognition, inputting the iris data directly into the frame might not achieve a good recognition effect. The unsteady-state iris in an uncertain state is prone to a situation in which the existing framework does not match the input iris data. When the iris data set is limited in size, the situation classification is limited, and altering the existing framework might reduce accuracy, thus greatly limiting the types of existing frameworks that can be used. The question of how to design the framework to better adapt to the iris data must be addressed.
2. In the study of feature expression and universal recognition, most methods normalize the data of different sensors and different environments by using algorithms such that the images can be identified under the same standard. However, not all processes are suitable for all images; the lack of suitability can cause unnecessary calculations and exclude certain images that can be identified but are not considered to be available irises because they do not meet the standard, possibly resulting in a lack of training of the multi-state iris model. Current research lack a universal process mechanism for the heterogeneous iris generated in a variety of different devices and environments. Because a single algorithm is prone to omission in multi-state features, many algorithms use multi-source feature expressions, an approach that requires in-depth understanding of the relationship between different types of features to avoid repeated calculation of iris features with large correlations and to improve the remoteness and discrimination of features.
3. With the limited size of the iris data set and the limitation of situation classification, it is necessary to judge the situation of recognition errors and establish a reverse correction mechanism for the recognition framework process. This mechanism not only avoids mechanical judgment by relying on a large amount of data to generate fixed indicators in statistical learning but also avoids the situation of cognitive loss caused by the inability to form new concepts when unknown situations occur in cognitive learning.

**Figure 4:**
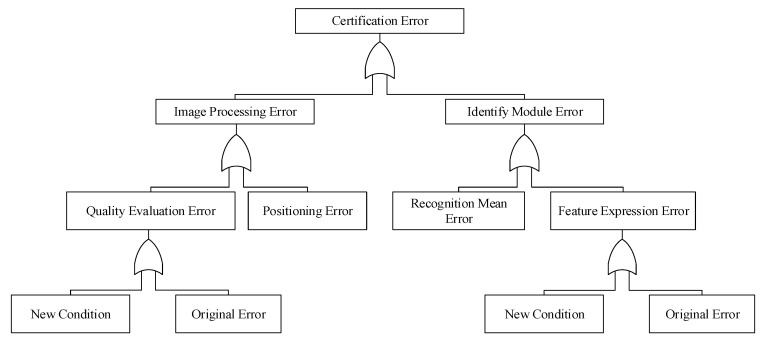
The fault tree of the certification model in the case of certification error.

The fault tree of the certification model in the case of certification error is shown in Figure 4.

According to the situation reflected in Figure 4 and the current problems, this paper proposes solutions for the following problems:

1. In face of the unsteady situation of heterogeneous irises produced by a variety of acquisition sensors and environments, the traditional processing method of a single source iris based on statistical learning or cognitive learning is not effective;
2. Traditional iris recognition divides the entire process into several statistically guided steps, which cannot solve the problem of correlation among various links;
3. The existing iris dataset size and situation classification constraints make it difficult to meet the requirements of learning methods in a single deep learning framework.

## 3. Evaluation Module

**Figure 5:**
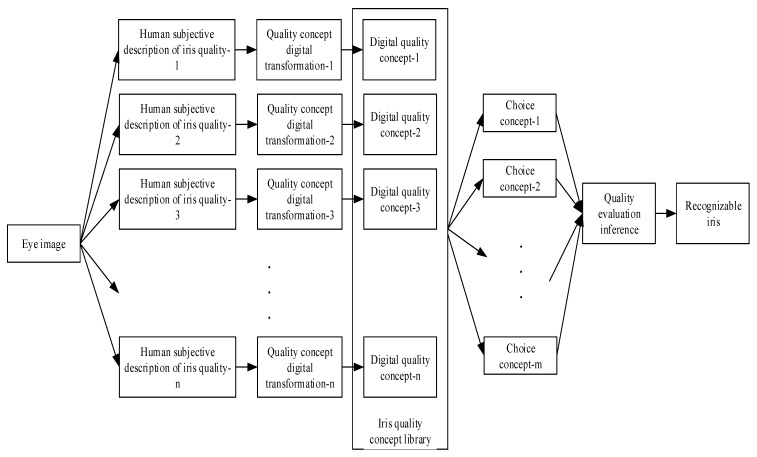
The whole structure of quality concept fuzzy inference.

The whole structure of quality concept fuzzy inference and the process of obtaining the results are shown in Figure 5.

The construction of iris quality concept fuzzy inference is mainly based on a pure fuzzy system structure. A set of iris quality evaluation processes common to different iris libraries is designed to determine the quality of the input image and input the identified recognizable iris into the subsequent recognition model. This system uses the iris quality knowledge concept construction mechanism to build the eye concept knowledge base and quality concept knowledge base, and complete the iris quality concept library. According to the actual requirements, the appropriate concept is selected as the use concept, and the iris that conforms to the selected concept rule is used as the qualified iris for the next iris recognition. This process is called iris quality inference.

### 3.1. Iris Quality Knowledge Concept Construction Mechanism

**Figure 6:**
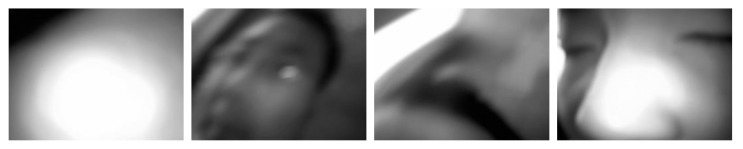
The example of an image that may be acquired before the eye is pointed at the camera.

The example of an image that may be acquired before the eye is pointed at the camera is shown in Figure 6.

When taking eye images, because of the fact that live detection is not considered, the difficulty of iris inspection is limited to the image confusion caused by the eyes before the camera is pointed at the time of human eye collection. Therefore, it is necessary to ensure that the image has an eye image inside. 

It can be seen from Figure 6 that it is necessary to establish the concept of the eye, and it is the purpose of establishing the eye concept knowledge base to ensure that some form of eye exists in the image. At the same time, a quality evaluation knowledge base is constructed based on the iris feature extraction and the demand of the recognition algorithm for iris quality. Because the iris quality evaluation itself is highly subjective, in the iris quality knowledge concept construction mechanism proposed in this paper, due to the limited number of eye images and in order to meet the applications of different devices and different environments, people can only form a subjective cognitive concept based on the objective laws of the relative relationship among the gray levels of various parts of the eye first according to all the initial training samples. People’s logical concepts are transformed into digital concepts of computers by means of image processing, data extraction, and designing the process in a way that determines thresholds are avoided as much as possible, and concept labels and label inference rules are formed. 

In the case where the collector is set as a living person, the formulation of the eye concept and the quality concept is continuously improved under the interaction mechanism between people and the system, transforming human subjective cognition into the digital concept of the computer system to ensure the universality of quality concept knowledge. The feedback mechanism must dynamically adjust according to the recognition results and the increase in external conditions, so that different feature extraction methods can get recognizable irises through identical evaluation reasoning process mechanisms.

**Figure 7:**
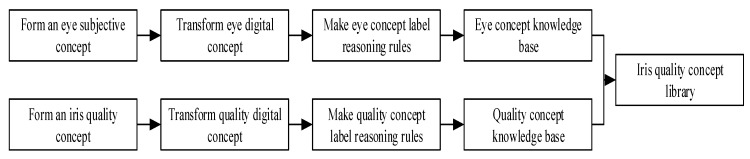
The operation process of iris quality knowledge concept construction mechanism.

The operation process of the iris quality knowledge concept construction mechanism is shown in Figure 7.

The operating steps of the iris quality knowledge concept construction mechanism are:

Step 1: Observe the state of the eyes of the training sample held by normal humans, and form the subjective concept of the eyes based on the visual impression of the eyes;

Step 2: Convert a subjective concept into a digital concept label through methods such as image processing and feature extraction;

Step 3: Formulate the inference rules for eye concept labels, combine multiple concept labels to form the concept of eyes, and build an eye concept knowledge base;

Step 4: In order to allow the unsteady state iris to be identified in different feature expression and recognition algorithms, reduce the probability of repeated extraction and design the iris quality knowledge base. Firstly, the subjective concept of iris quality is formed by observing the feelings of training samples held by normal humans according to the demand for iris quality;

Step 5: Use methods such as image processing and feature extraction to convert subjective concepts about quality into digital concept labels;

Step 6: Formulate inference rules for quality concept labels, combine multiple concept labels together to form the concept of the image of iris recognition, and build the iris quality concept knowledge base;

Step 7: Combine the iris quality knowledge base with the eye concept knowledge base to form the iris quality concept library.

### 3.2. Example of Iris Quality Concept Fuzzy Inference

This section uses a specific example to show how to use the iris quality knowledge concept construction mechanism to build the iris quality concept library when the iris conditions meet the prerequisites of this paper, and in this example, all iris knowledge is selected for iris quality inference to get recognizable irises and realize the versatility of collecting iris for different iris collectors.

#### 3.2.1. Eye Concept Knowledge Base and Eye Inference

Because the acquisition situation before the glasses are aligned with the camera is unpredictable, and to ensure that the eye concept of the unsteady state iris can be universal, the establishment of the initial eye concept knowledge base adopts the form of subjective morphological description. First, the eye image needs to be processed to facilitate the transformation of the subjective morphological description of the human eye into the objective data form of the computer. The specific process is as follows:

The first step: process the eye image, and segment the corresponding parts to extract the eye concept:

1. **Pupil region segmentation:** The pupil is the most critical part of the eye. The pupil concept is established to facilitate the location of the iris. Therefore, it is necessary to narrow the pupil in the image first. The collected iris image is processed by the non-linear function (1). Equation (1) is as follows:

(1) $$T=e^{\left(\frac{f(x,y)}{255}-0.5\right)}$$

where *e* represents the base number in the exponential function, and f(x,y) represents the gray value of the point with coordinate (x,y) in the image.

Because the captured image is set as a grayscale image, the grayscale range of the image is [0,255], so the range of the resulting value *T* is [$e^{-0.5}$, $e^{0.5}$], and image segmentation is performed with 1 as the cutoff point. Avoid the difficulty of threshold selection due to the large gray range. According to the law of the gray value of the eye, no matter how the external light changes, the gray value of the pupil is generally smaller than the iris sclera eyelid and other areas, so the pupil value should be in front of the overall range (less than 1), so take the gray value of the result value at [$e^{-0.5}$, 1]. If most gray values are greater than 1 or only a few are less than 1, it means that there are no eyes, or the light intensity of the eyes is too dark or too bright, so that the pupils in the eye image are not obvious. The image can be segmented by formula 1, which means that the image itself has no extreme conditions, that is, the gray value difference between the pupil and iris is relatively normal. The image noise interference is removed through the open operation [29], and a grayscale image with pupils is obtained as the eye discrimination image.

2. **Binary image of pupils:** Design formula 2 to process the eye discrimination image.

(2) $$f_{1}(x,y)=\{\begin{array}{cc} f(x,y)-0.5\times z & f(x,y)>z\\ f(x,y)-0.5\times z-\min & f(x,y)\leq z \end{array}$$

Among them, *z* represents the average value of all points in the eye discrimination image whose gray value is not 0 or 255; $f_{1}(x,y)$ represents the result value of the gray value of the point with the coordinate (x,y) in the image after being processed by formula 2; min represents the minimum gray value (the gray value of the image is not 0 or 255) of all points in the eye to discern. Set the gray values of the points where the value of $f_{1}(x,y)$ is less than or equal to 0 to 0, and set the gray values of the other points to 255 to obtain a binarized image of the pupil, which contains a connected area related to the pupil.

Design idea and purpose of Equation (2):

(1) It can be seen from Figure 5 that although the pupils are identified in the eye discrimination image, the pupils are still incomplete due to light and eye hair. Therefore, in order to better determine the position of the pupil, it is necessary to reflect the pupil rules more accurately, so binarization is required.
(2) By lowering the eyes to determine the overall gray value of the image, while making the low gray value closer to 0, the relative relationship between the gray values is not changed, and the pupil site is better found. The reason for subtracting half of the average gray value is that under the unstable iris, the specific situation of the gray value of each image is uncertain, and it is not safe to use fixed parameters. Therefore, a non-fixed value related to the nature of the image is needed as the parameter for function design. Because the gray value of the pupil itself is relatively small, the gray value will be less than the average value in most cases, but the unpredictable situation in the unsteady iris is more complicated, and the gray value of other non-pupil parts cannot be guaranteed. Subtracting the average value may cause points that are larger than the grayscale average value to be suppressed too much, affecting the appearance of the pupils. Therefore, only half of the average grayscale value is subtracted in the function design.
(3) For points where the gray value is lower than the average value, subtracting the minimum gray value ensures that the pupil can be separated to the greatest extent. Then the difference between pupil and eye hair can be distinguished, and then as much of the pupil connected area can be identified as possible.

Therefore, the design idea of formula 2 is to as much of the pupil connected area as possible and eliminate the interference of the non-pupil area.

**Figure 8:**
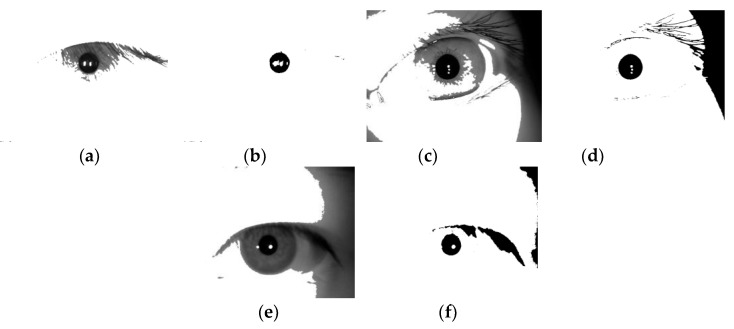
The eye discrimination image. (**a**) Eye discrimination image collected by the device in Figure 2b, (**b**) the image of Figure 2a corresponds to the binarized image of the pupil, (**c**) eye discrimination image collected by the device of Figure 2d, (**d**) the image of Figure 8c corresponds to the binarized image of the pupil, (**e**) eye discrimination image collected by the device of Figure 2f, (**f**) the image of Figure 8e corresponds to the binarized image of the pupil.

The eye discrimination image and the corresponding binarized image of the images collected by different types of devices after this step are shown in Figure 8 (corresponding to the image of the device of Figure 2).

3. **Preliminary determination of the pupil area:** The location of the pupil connected area boundary is found by performing a canny edge detection [23] operation and Hough circle detection [24] operation on the binarized image of the pupil, and the pupil center and radius are determined.

(1) It can be seen from Figure 8 that although the shape of the pupil connection area is irregular and complete, it can be regarded as a circle. Therefore, in order to reduce the calculation amount, the existing circle detection technology is used to fit the pupil connection area.
(2) During the detection, due to the unpredictability of the unsteady state iris, the circle radius of the pupil connection area cannot be set in a fixed area. The image dimension of the image used in this paper is 640 × 480. In order to reduce the computational complexity, the image dimension is reduced to 160 × 120 by the majority statistical pooling method to form a reduced- dimensional image.

Majority statistical pooling method: The image is divided into 160 × 120 sub-blocks according to the 4 × 4 sub-blocks. The number of points with a gray value of 0 in each sub-block is counted, and if it is greater than 8, the point is considered to be in a small gray value region, and the gray value of the corresponding point in the reduced-dimensional image is set to 0, otherwise set to 255. The reason for using 4 × 4 dimensional sub-blocks is because according to the physiological iris features, the radius of the iris is about 1–2 times the pupil radius. Therefore, the iris acquisition image with a width of 480 dimensions can accommodate up to 6 times the pupil radius, that is, 80 dimensions, and after being reduced 4 times, it is 20 dimensions. Six times of twenty is 120, so the 160 × 120 dimensional reduced image can meet the pupil requirements in non-extreme cases.

Because the threshold of circle detection is not fixed in the pupil binarized image, multiple circle centers and radii may be detected in an image. In an unstable iris, the pupil position and radius cannot be completely determined. Therefore, these detected possible circle centers and corresponding radii are used as eye concept label candidate data.

**Figure 9:**
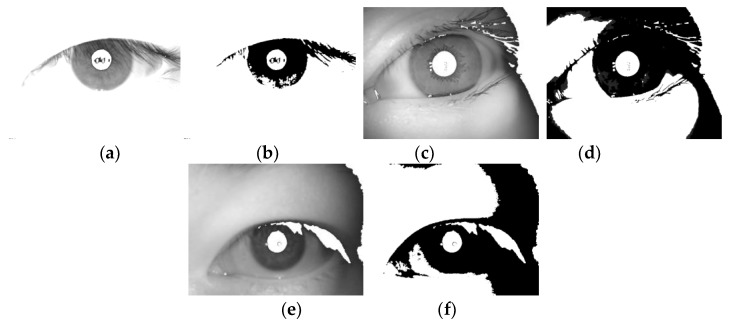
The transition processed image. (**a**) The transition processed image of Figure 2b, (**b**) the image of Figure 8a corresponds to the connected area, (**c**) the transition processed image of Figure 2d, (**d**) the image of Figure 8c corresponds to the connected area, (**e**) the transition processed image of Figure 2f, (**f**) the image of Figure 8e corresponds to the connected area.

4. **Iris connected area:** Use the detected center and radius to make candidate data images, and then extract concept labels. The position of the point where the gray value is 0 in the pupil binarized image correspondes to the original image, and the gray value of the point in the eye discrimination image is set to 255 to obtain a transition processed image. Because the iris region should be a small gray value region in the transition processed image, the same processing is performed on the processed image using Equation (2) to obtain an image containing the iris connected region. The transition processed image of each part corresponding to the image in Figure 8 and the image of the connected area of the iris are shown in Figure 9.

**Figure 10:**
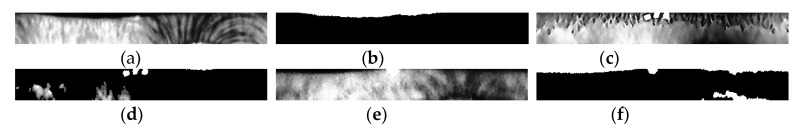
The example of a normalized image. (**a**) Normalized enhanced image of Figure 2b, (**b**) normalized image of Figure 9b, (**c**) normalized enhanced image of Figure 2d, (**d**) normalized image of Figure 9d, (**e**) normalized enhanced image of Figure 2f, (**f**) normalized image of Figure 9f.

Using the Daugman rubber band method [30], the center of each pupil is detected as the polar point, and the radius is 2 times the polar axis, and the radius of the iris connected area and the original image are all doubled to 2 The annular area between the double-radius lengths is transformed into a normalized image of 512 × 64 dimensions and the texture is highlighted by equalizing the histogram [31] to form a normalized enhanced image. At the same time, the normalized processing of the iris connected area is also performed. The example of the normalized enhanced image of Figure 2 and the normalized image of the iris connected area (image of Figure 9) correspond to the image of Figure 10.

**Figure 11:**
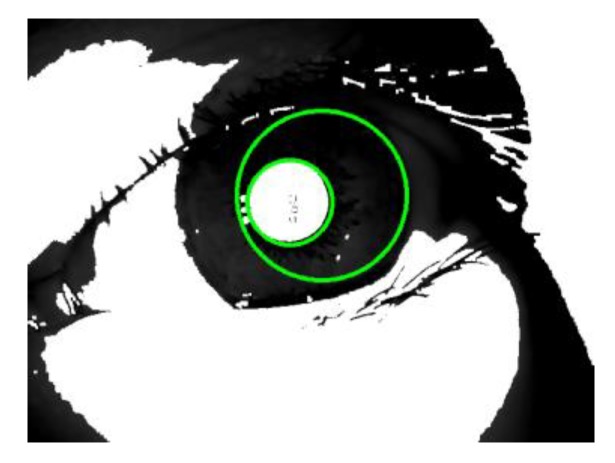
Example of the circle range of the detected different center and radius in the iris connected area.

The image in Figure 10 is used as the basis of the obtained center and radius data to construct knowledge in the eye concept knowledge base. After the collected images are processed, the concept labels in the eye concept knowledge base are constructed, and the concept label rules are developed to convert human subjective concepts into digital concepts and form judgment rules, which together serve as the knowledge reserve. The process is as follows:

1. **Pupil:** According to the iris connected area image, the inside of the pupil is a white area with a gray value of 255 similar to a circle. The circle range of the detected center and the radius in the iris connected area are different, as shown in Figure 11.

It can be seen from Figure 11 that the gray value of a point inside a circle that can well reflect the pupil range is mostly 255. The design of the conceptual label of the pupil should satisfy this phenomenon.

Therefore, the subjective concept of the pupil is that most points in the pupil satisfying the circled range are white points, and the white points are evenly distributed in each part.

The process of turning subjective concepts into digital concepts is as follows:

(1) In the image of the connected area of the iris, divide the pupil range circled by a detected center radius into four equal parts;
(2) Calculate the number of points with a gray value of 255 in these four parts, and calculate the proportional value $K_{i}$ of the points in the area;
(3) Calculate the variance value *T* of the proportional value $K_{i}$ of the four parts.

The four proportional values $K_{i}$ and the variance value *T* are the numerical concept labels of the subjective concept.

The concept label rule of the digital concept label is expressed by Formula 3. Formula 3 is as follows:

(3) $$\sum_{i=1}^{4}(1-K_{i})<\eta \;T\to \mu$$

Among them: η and μ are real numbers close to 0, which are set according to the collector device and the specific environment.

When the sum of the difference between the four proportional values and 1 is infinitely close to 0 and the variance value is close to 0, it means that there are pupil-like parts in the circled range.

**Figure 12:**
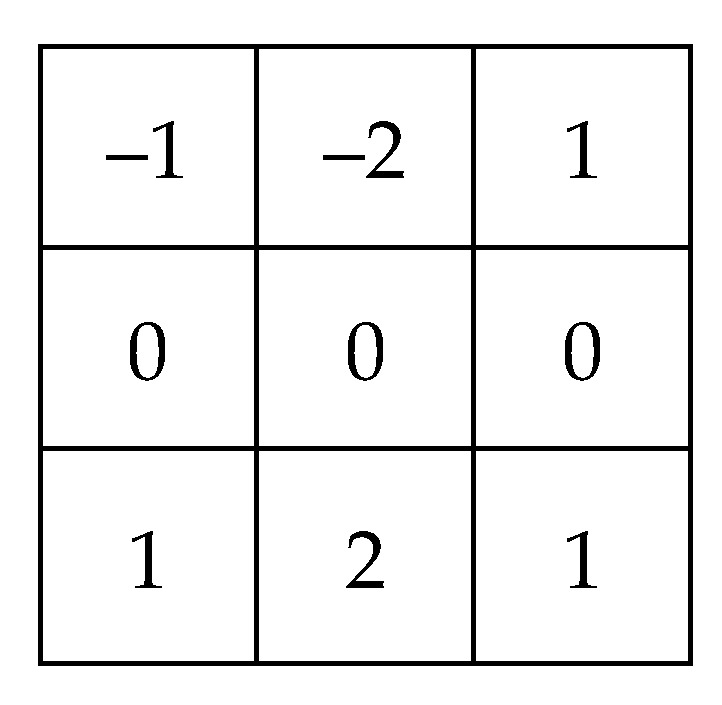
Sobel operator.

2. **Iris:** It can be seen from Figure 9 that there is a certain amount of iris around the pupil, so it should be an iris area around the pupil that is accurately circled, and the iris area is considered from the perspective of vision to consist of irregular and uniformly distributed textures, In terms of an image, it is visually more inclined to the vertical form, so a Sobel operator that can extract vertical features is used, which is shown in Figure 12.

**Figure 13:**
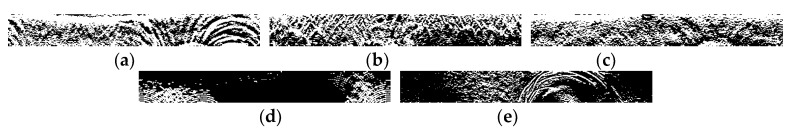
Example of salient images with and without an iris image. (**a**) Salient image of Figure 2b, (**b**) salient image of Figure 2d, (**c**) salient image of Figure 2f, (**d**) example of eyeless highlight image, (**e**) example of salient image with incorrect pupil delineation.

The Sobel operator is used to perform the texture highlighting on the normalized image, and the gray value of the points whose gray value is lower than 0 is set to 0, and the gray value of which is greater than 0 is 255. The example of salient images with and without the iris image are shown in Figure 13.

It can be seen from Figure 13 that after the iris image is highlighted again, the points with a gray value of 255 are distributed throughout the highlighted image. Images with no eyes or no iris have fewer point distributions with a gray value of 255, which is used as a subjective concept for the iris.

**Figure 14:**
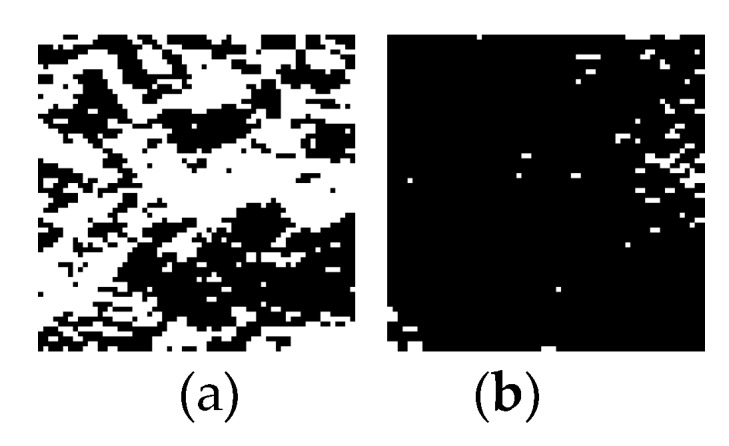
The detection image of the iris part and the detection image of the non-iris part. (**a**) Detection image of iris area, (**b**) detection image of non-iris area.

The process of turning subjective concepts into digital concepts is as follows:

(1) In order to reduce the amount of calculations, the normalized image of the iris connected area image is intercepted, and the 512 × 64 dimension image is divided into eight 64 × 64 dimension images on average. The intercepted coordinate points of the image with the smallest gray value among the 8 images is recorded;
(2) The outstanding image is equally divided into eight 64 × 64 dimensional images, and the image at the same position as the intercepted coordinate point recorded in 1) is selected as the iris detection image. The detection image of the iris part and the detection image of the non-iris part are shown in Figure 14.
(3) The minimum pooling method is used to minimize the detection image in 2 × 2 dimensions [27] to form a 32 × 32 dimensional pooling detection image. In order to better reflect the irregular and black-and-white form of the iris, the Hamming distance [28] is used for representation. Set the points with a gray value of 255 to 1 and the points with a gray value of 0 to 0 to form 32 binary sequences. Calculate the Hamming distance between each adjacent sequence, a total of 31 values, numbered $H_{1}-H_{31}$.

The 31 Hamming distances are the digital concept labels for this subjective concept. The concept label rule of the digital concept label is expressed by formula 4, which is as follows:

(4) $$\sum_{i=1}^{31}(1-H_{i})<\alpha$$

Among them: α is a real number close to 0, which is set according to the collector device and the specific environment.

In each row, 1 is distributed irregularly and exists, and the ideal situation is that the Hamming distance between each row will be close to 1. Therefore, when the sum of the difference between the 31 Hamming distances and one is close to 0, it means that there are iris-like parts in this area. 

#### 3.2.2. Quality Concept Knowledge Base and Quality Inference

In the example presented in this paper, because of the unpredictability of the unsteady iris, it is difficult to ensure that a single fixed index can be used to evaluate the quality of the iris. Iris images must be excluded in extreme cases, as many images as can be recognized must be kept, and the scope of iris recognition must be expanded. In the process of digitizing the concept, based on the premise that there are eyes in the image and in the case of an unsteady state without determining the expression and recognition of iris features according to a person’s intuitive impression of the iris, an image that can be recognized as the iris should not be too blurred or excessively squinted. Therefore, design ambiguity and direct vision for iris quality evaluation, excluding irises that cannot be identified, improve the detection rate of unsteady irises, and pave the way for the expression and recognition of unsteady irises afterwards.

The process of transforming subjective concepts into digital concepts and setting rules for concept labels is as follows:

1. **Image blur:** Determine the center of the pupil in the eye as the pole of the polar coordinates, and convert all the annular areas between 1 times the radius length and 2 times the radius length in the image into a normalized image of 512 × 64 dimensions. A 256 × 32 dimensional intercepted image is taken from the upper left corner as a blur test image.

Set the points in the normalized image whose gray value is not 0 or 255 as the feature points. According to formula 5, formula 6 obtains the image blur degree. Formula 5:

(5) $$H_{i}=|F(x_{i},y_{i}+1)-F(x_{i},y_{i})|+|F(x_{i},y_{i}-1)-F(x_{i},y_{i})|+|F(x_{i}+1,y_{i})-F(x_{i},y_{i})|+|F(x_{i}-1,y_{i})-F(x_{i},y_{i})|$$

Among them: $F(x_{i},y_{i})$ represents the gray value of the *i-th* feature point with coordinate $\left(x_{i},y_{i}\right)$ in the normalized image; $F(x_{i},y_{i}+1)$ represents the gray value of the adjacent point above the feature point with coordinate $\left(x_{i},y_{i}+1\right)$ in the normalized image; $F(x_{i},y_{i}-1)$ represents the gray value of the adjacent point above the feature point with coordinate $\left(x_{i},y_{i}-1\right)$ in the normalized image; $F(x_{i}+1,y_{i})$ represents the gray value of the adjacent point to the right of the feature point with coordinate $\left(x_{i}+1,y_{i}\right)$ in the normalized image; $F(x_{i}-1,y_{i})$ represents the gray value of the adjacent point to the left of the feature point with coordinate $\left(x_{i}-1,y_{i}\right)$ in the normalized image; $H_{i}$ represents the sum of the absolute value of the difference in gray value between the *i-th* feature point with coordinate $\left(x_{i},y_{i}\right)$ in the normalized image and the gray values of the four adjacent points on the upper, lower, left, and right sides.

Formula 6 is as follows:

(6) $$D=\frac{\sum_{i=1}^{s}H_{i}}{s}$$

Among them: *D* represents the normalized image blur; *s* represents the number of feature points in the normalized image.

According to the specifications of different collectors, when a clear threshold *p* is set, and *D* is greater than or equal to *p*, it means that the image collected by the collector is clear, and the iris image processing can continue. When *D* is smaller than *p*, it means that the image collected by the collector is blurred, and the iris image processing cannot be continued.

2. **Image visibility:** The center point of the pupil is the center point. The gray value of the point in the grayscale image of the iris that is more than 5 times the radius length and less than 1 time the radius length is set to 255, and the gray value of the other points is unchanged. The direct view of the image according to the four parameters obtained by formula group 7 is calculated, and the dimensions of the grayscale image of the iris is set to to *M* × *N*, where *M* represents the length and *N* represents the width (Formula 7):

(7) $$G_{1}=\frac{\sum_{i=1}^{N}\left|\mathrm{sgn}(f_{2}(M,y1_{i})-255)\right|}{N}\;G_{2}=\frac{\sum_{i=1}^{N}\left|\mathrm{sgn}(f_{3}(0,y2_{i})-255)\right|}{N}\;G_{3}=\frac{\sum_{i=1}^{M}\left|\mathrm{sgn}(f_{4}(x1_{i},N)-255)\right|}{M}\;G_{4}=\frac{\sum_{i=1}^{M}\left|\mathrm{sgn}(f_{5}(x2_{i},0)-255)\right|}{M}$$

Among them: $f_{2}(M,y1_{i})$ represents the gray value of the *i-th* point of the coordinate $\left(M,y1_{i}\right)$ on the rightmost boundary; $G_{1}$ represents the ratio of the sum of the number of points with gray values not equal to 255 on the rightmost boundary of the image to the dimension *N*; $f_{3}(0,y2_{i})$ represents the gray value of the *i-th* point of the coordinate $\left(0,y2_{i}\right)$ on the leftmost boundary; $G_{2}$ represents the ratio of the sum of the number of points with gray values not equal to 255 on the leftmost boundary of the image to the dimension *N*; $f_{4}(x1_{i},N)$ represents the gray value of the *i-th* point of the coordinate $\left(x1_{i},N\right)$ on the lowermost boundary; $G_{3}$ represents the ratio of the sum of the number of points with gray values not equal to 255 on the lowermost boundary of the image to the dimension *M*; $f_{5}(x2_{i},0)$ represents the gray value of the *i-th* point of the coordinate $\left(x2_{i},0\right)$ on the uppermost boundary; $G_{4}$ represents the ratio of the sum of the number of points with gray values not equal to 255 on the uppermost boundary of the image to the dimension *M*; sgn is a symbolic function that represents the sign of the return parameter. The direct view of the image is determined according to formula 8:

(8) $$Zs=\mathrm{sgn}((\mathrm{sgn}(G_{1}-q)+\mathrm{sgn}(G_{2}-q)+\mathrm{sgn}(G_{3}-q)+\mathrm{sgn}(G_{4}-q))+3)$$

Among them: *q* represents the discrimination threshold, which is set according to the specifications of different collectors.

Zs represents the value of image visibility. If Zs = –1, it is determined that the image visibility of the image cannot continue to perform iris image processing. If Zs = 1, the image visibility of the image is determined to continue iris image processing.

According to the image blur and image direct vision, the iris quality concept knowledge base (poor quality iris knowledge base in this paper) is formed, and the concept of eyes is combined to jointly build the iris quality concept library in the example.

### 3.3. Feedback Learning Mechanism of Quality Fuzzy Inference

Uncertainty caused by iris instability, limitations on the cognition of iris quality and digital methods, and restrictions on the number and types of initial irises all lead to inadequate initial concept settings, which lead to incorrect certification results. Under the prerequisites of this paper, the recognition error caused by the iris exceeding the prerequisites of the recognition model is ruled out. In this paper, the cause of the certification error is identified only as a problem in the certification process.

When building the knowledge base, there are some drawbacks for the following reasons:

1. The subjective concept of the human eye is limited by the accumulation of human knowledge. The subjective impressions obtained only by visual observation of the data possessed are not necessarily completely accurate.
2. The method of transforming the subjective concept into a digital concept can digitally represent the subjective description of the iris only adequately; however, this does not mean that the means of expression must be the best and unique.
3. As the number of acquisitions increases and the external conditions of the acquisition and the needs of the iris change, the perception of the subjective concept of the iris may change.

Because the iris quality evaluation index is highly subjective and the application environment of this article is an unpredictable unsteady-state iris, it is difficult to accurately modify the quality standard in the traditional function type judgment method, and only the final recognition result can reflect the performance of the quality concept. Therefore, the feedback dynamic learning mechanism proposed in this paper is initiated every time a misidentification or a new recognition requirement occurs. The purpose is to analyze the cause of the error, adjust or expand the quality concept, and improve the accuracy of the iris quality evaluation, thus making the iris more adaptable to the recognition method, thereby improving the accuracy of iris recognition. After the feedback dynamic learning mechanism is initiated, humans with professional knowledge will assist in analyzing the causes of the errors and then propose appropriate solutions for improvement to meet most of the principles. Except for a very few extreme cases, most images can be correctly identified.

The operating steps of the quality learning system and feedback dynamic learning mechanism are as follows:

1. When it is determined that the quality concept should be adjusted, analyze the existing quality concept labels, each label setting process one by one, and whether the process settings are standardized; then, record if there are irregularities.
2. Modify the parameter settings in the original process appropriately, observe the modified recognition results, and then analyze the relationship between each quality concept label and the standardization of the settings to discover loopholes and record them; analyze various recording situations, modify existing quality concept labels (for human subjective concepts, digital expression of subjective concepts, etc.), quality concept label representation methods, and image processing processes to correct the quality concept labels.
3. When the recognition environment changes and it is necessary to expand the concept library, analyze the new environment and conditions, add new quality concepts, and expand the quality concepts (by creating new concepts and new digital expression methods).

The revised quality concept inference system requalifies and recognizes the new image and observes the results again. If the recognition is correct, the correction is completed. An incorrect recognition indicates that the correction is insufficient. Repeat steps 1–3 and continue to modify the quality concept until no iris image is available or it is determined that there is an error in the certification module.

## 4. Certification Module

Under the premise that the amount of data is small and that various states cannot be accurately classified, the iris can only be identified based on mixed data. It is necessary to design a neural network structure that can recognize this situation, which can achieve lightweight feature label settings and heterogeneous authentication in the case of one-to-one certification. Therefore, the neural network structure should not be complicated. In this regard, this paper uses a convolutional neural network [32] with a lightweight iris certification layer structure.

### 4.1. Iris Processing 

**Figure 15:**
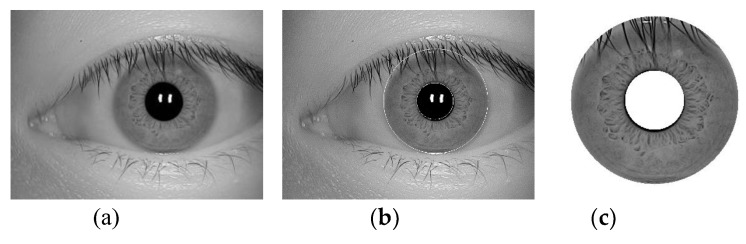
The image of each stage of positioning. (a) Quality qualified image, (**b**) positioning image, (**c**) segmentation image.

**Figure 16:**
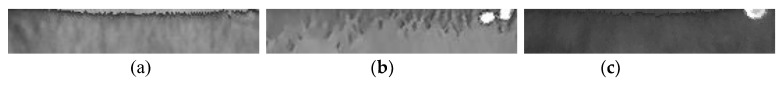
Examples of iris recognition areas of different iris libraries. (**a**) Iris recognition area of Figure 2b, (**b**) iris recognition area of Figure 2d, (**c**) iris recognition area of Figure 2f.

The images of each stage of positioning are shown in Figure 15. Examples of iris certification areas of different iris libraries are shown in Figure 16.

Iris images must be processed prior to feature extraction. In this paper, the normalized iris image has dimensions of 360 × 64, and the 180 × 32 dimensional area is taken from the upper left corner as the recognition area. In the actual iris acquisition process, under external environmental influences such as illumination, defocusing, and the deflection and change of the collector state, many states of iris presentation might occur. To ensure the efficiency of shooting and recognition, the qualified standard of the iris quality evaluation method for continuous frames is low, and only extreme conditions with poor iris quality are removed.

### 4.2. Multi-Source Multi-Feature Fusion Mechanism and Heterogeneous One-to-One Certification

The multi-source multi-feature fusion mechanism for iris feature extraction is established. The image of the iris recognition area is processed by a smoothing algorithm and a texture highlighting algorithm. Each iris image passes through 12 layers of an image-processing network consisting of convolutional layers, pooling layers, ReLU layers, and an expansion layer. Finally, each iris image forms 15 expanded parameters and a total of 45 expanded parameters in the expansion layer. The expanded parameters of the three images are fused by average fusion through the feature fusion layer to form 15 recognition parameters. 

#### 4.2.1. Feature Expression

The traditional single-angle statistical learning method encounters difficulty in collecting the multi-state iris. Therefore, a multi-source method is used to express the features from multiple angles. Smooth filtering and texture highlight filtering are applied. It is better to find a stable region that can reflect the texture change relationship inside the iris and to suppress the influence of illumination and defocusing on the feature expression as much as possible.

**Figure 17:**
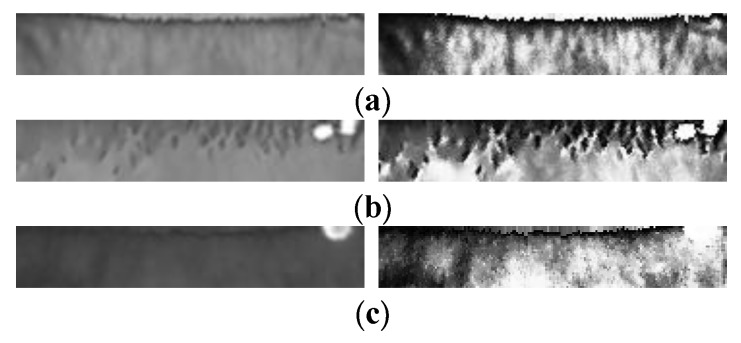
An example of the filtered image. (**a**) Gaussian filtering of Figure 6a, (**b**) equalization histogram of Figure 6a, (**c**) Gaussian filtering of Figure 6b, (**d**) equalization histogram of Figure 6b, (**e**) Gaussian filtering of Figure 6c, (**f**) equalization histogram of Figure 6c.

This paper uses Gaussian filtering [33] (smoothed) and the equalization histogram [34] (highlighted) as examples to explain the image-processing network. An example of the filtered image in Figure 16 is shown in Figure 17.

Each image is processed by the same image-processing method. The purpose of image processing is to highlight the iris texture as much as possible, so that the value of the recognition parameters reaches a relatively large level, in order to facilitate the use of the certification function. Therefore, some common convolution kernels can be used to highlight the iris texture. This paper uses the gradient Laplacian convolutional kernel [35], Sobel convolutional kernel [36], gradient convolutional kernel [37], and eight-neighborhood convolution operator with a center of nine as an example and combines them.

The specific steps of the image-processing module are listed as follows:

**First step:** Input an iris image into the first convolutional layer. A gradient Laplacian convolution kernel is used. After image convolution, the image is converted into 2 × 2 maximum pooling [38] in the first pooling layer, resulting in a 90 × 16 dimensional image, thus thinning the pooled image through the Softplus function [39] in the first ReLU layer.

The Softplus function is shown in Equation (9):

(9) $$Softplus(x)=\log(1+e^{x})$$

where *x* represents the gray value of each point of the first pooling image, and Softplus(x) is the resulting value of each point of the first ReLU image. 

**Figure 18:**
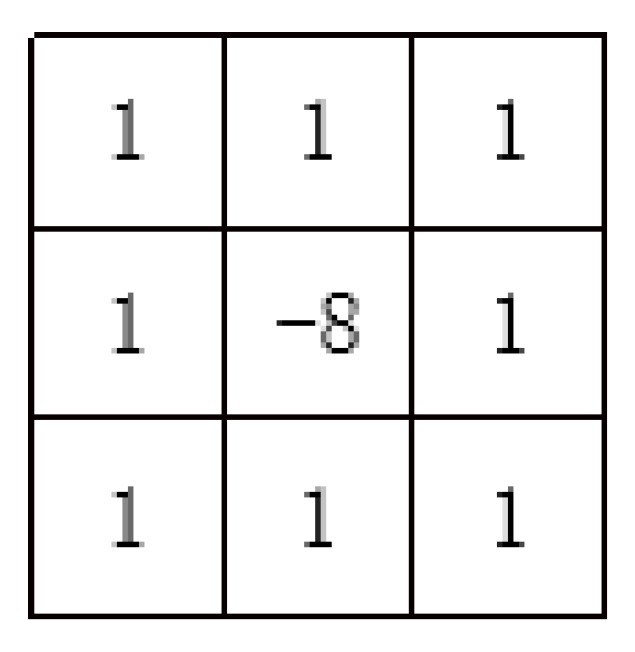
Gradient Laplacian convolutional kernel of the first convolutional kernel.

The convolutional kernel of the first convolutional layer is shown in Figure 18. 

**Figure 19:**
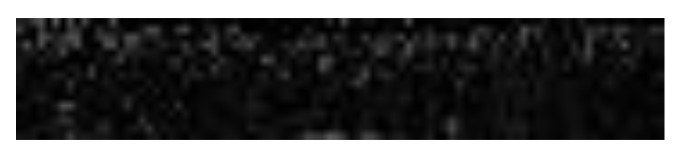
An example of the processed image formed in the first step.

Finally, the result in the first ReLU layer is one processed image. For the original image (taking Figure 2b as an example), an example of the processed image formed in the first step is shown in Figure 19.

**Second step:** The second convolutional layer uses three convolution kernels, namely the gradient Laplacian convolution kernel, horizontal Sobel convolution kernel, and vertical Sobel convolution kernel. The gradient Laplacian convolution kernel is the same as the first convolution layer. After a ReLU image is convolved, three convolutional images are formed. The image is converted into a 45 × 8 dimensional image by 2 × 2 maximum pooling in the second pooling layer and the Softplus function in the second ReLU layer. The second ReLU layer uses the same Softplus function as the first ReLU layer to perform thinning operations on the pooled images in the second pooling layer.

**Figure 20:**
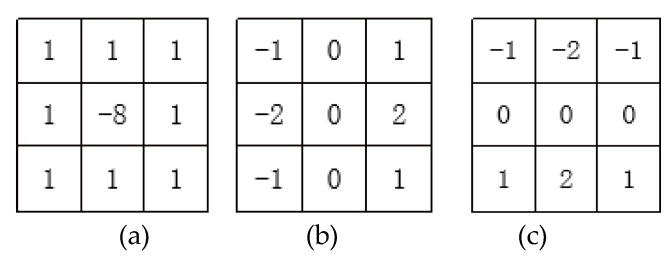
Convolutional kernels of the second convolutional layer: (**a**) gradient Laplacian convolutional kernel, (**b**) horizontal Sobel convolutional kernel, and (**c**) vertical Sobel convolutional kernel.

The convolutional kernels of the second convolutional layer are shown in Figure 20.

**Figure 21:**
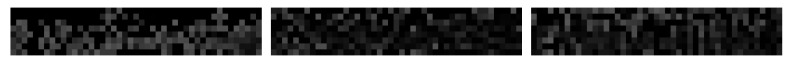
An example of three processed image formed in the second step.

The result in the second ReLU layer is three processed images. An example of the three processed images (taking the image processing process in Figure 9 as an example) formed in the second step is shown in Figure 21.

**Third step:** The third convolutional layer uses five convolution kernels, namely the gradient Laplacian convolution kernel, horizontal Sobel convolution kernel, vertical Sobel convolution kernel, horizontal gradient convolution kernel, and vertical gradient convolution kernel. The gradient Laplacian convolution kernel is the same as the first convolution layer, the horizontal Sobel convolution kernel is the same as the second convolution layer, and the vertical Sobel convolution kernel is the same as the second convolution layer. After convolving three second ReLU images, 15 convolutions are formed. In the third pooling layer, the image is converted into a 22 × 4 dimension image by 2 × 2 maximum pooling, and via the Softplus function in the third ReLU layer, the third ReLU layer uses the same Softplus as the first ReLU layer function to perform the thinning operation on the pooled image in the third pooling layer.

**Figure 22:**
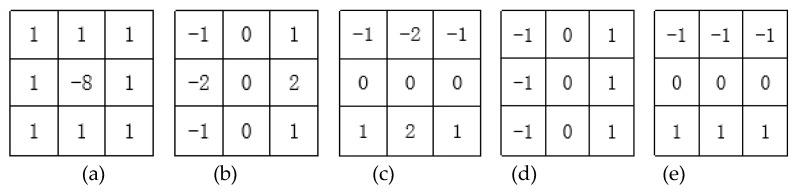
Convolutional kernels of the third convolutional layer: (**a**) gradient Laplacian convolutional kernel, (**b**) horizontal Sobel convolutional kernel, (**c**) vertical Sobel convolutional kernel, (**d**) horizontal gradient convolutional kernel, (**e**) vertical gradient convolutional kernel.

The convolutional kernels of the third convolutional layer are shown in Figure 22.

**Figure 23:**
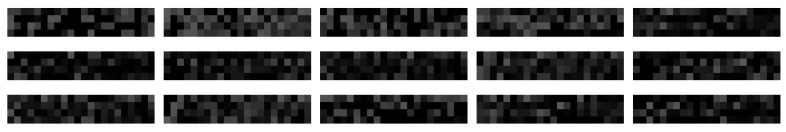
An example of fifteen processed image formed in the third step.

The result in the third ReLU layer is 15 processed images. An example of the 15 processed images (taking the image processing process in Figure 11 as an example) formed in the third step is shown in Figure 23.

**Figure 24:**
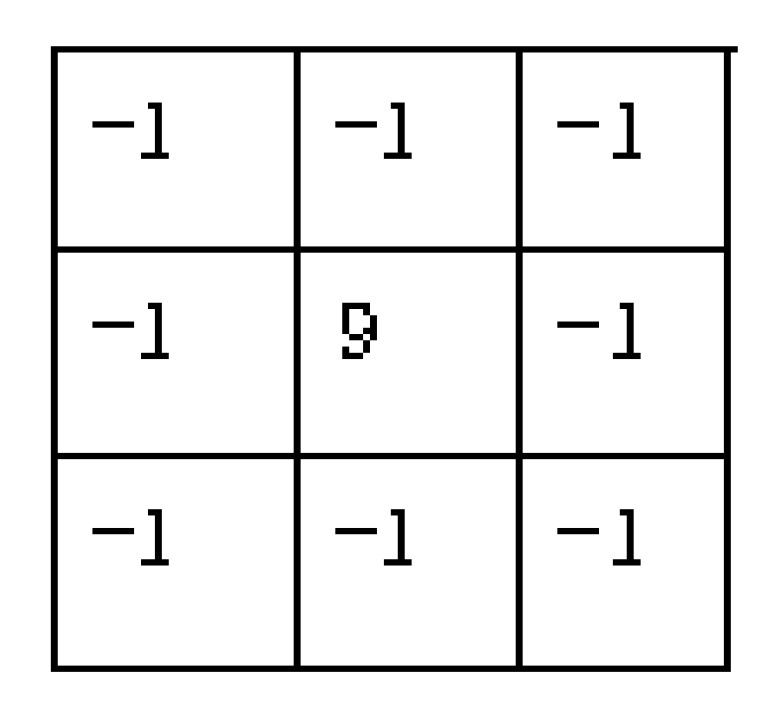
Convolutional kernels of the fourth convolutional layer.

**Fourth step:** Input image of the third ReLU layer into the fourth convolutional layer. The purpose is to sharpen the edges of the image and enhance the local image contrast. An 8-neighborhood convolution operator with a center of nine is used to convolve the image. A neighborhood convolution operator with a center of nine is shown in Figure 24.

**Figure 25:**
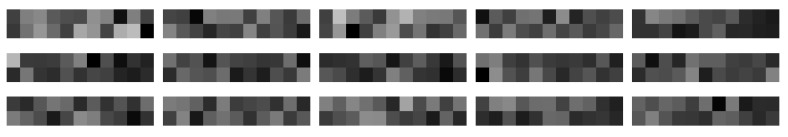
An example of fifteen processed image formed in the fourth step.

The processed image is converted into an 11 × 2 dimension image by 2 × 2 maximum pooling, with a total of 15 images. An example of the 15 images (taking the image processing process in Figure 13 as an example) formed in the fourth step is shown in Figure 25.

The average gray value of the 15 images is read and input into the expanded layer, and the image feature is converted into the data feature; the result of the expanded layer is 15 numbers, and the 15 numbers are the expanded parameters of an iris image. 

The three iris images (the original image, the smooth image, and the texture-emphasized image) are processed through the above steps, and the expanded parameters of the three images are collected. Three sets of the 15 expanded parameters can be obtained in the expanded layer. The average of the three numbers at the same position in the three sets as that of the image’s recognition parameters is calculated. The recognition parameters consist of 15 numbers.

#### 4.2.2. Iris Certification

After obtaining a sufficiently large recognition parameter, according to the iris training samples, it is necessary to set the category labels of different categories and set the number of training irises to *m*. Because these images are all iris components, the identified recognition parameters can represent the iris feature. The parameters of the certification function are set based on the training data currently held. The training iris is used as a test object, and a single category label is set according to the distribution of the data; as the amount of iris data increases, an analysis of certification error conditions is performed. The parameters of the certification function are first determined to ascertain whether they need to be updated under the effect of the feedback learning mechanism. If it is determined that they need to be updated, then the parameters are updated.

The steps for iris recognition are given as follows:

1. **Set the iris information entropy and recognition parameter labels:** Calculate the average value $L_{i}$ of the recognition parameters of each training iris (the average value of the *i-th* recognition parameter in the training iris), calculate the probability $p_{i}$ that the *i-th* recognition parameter is less than $L_{i}$ in all training images, and calculate the information entropy of each recognition node under different conditions according to Formula 10.

(10) $$H1_{i}=-p_{i}\times \log(p_{i})\;H2_{i}=-(1-p_{i})\times \log((1-p_{i}))$$

where $H1_{i}$ represents the information entropy with a recognition parameter less than $L_{i}$, and $H2_{i}$ represents the information entropy with a recognition parameter greater than $L_{i}$. Finally, $H1_{i}\times p_{i}+H2_{i}\times (1-p_{i})$ is used as the category label of the iris.

2. **Calculate the matching value** HD **between the template feature information and test feature information**: The objective of the recognition function is to find a category with sufficient discrimination and move the test iris feature as close as possible to the category label and observe whether it matches.

We set each recognition parameter of the test iris to $f_{i}$ (*i-th* recognition parameter) and calculate the information offset $Z_{i}$ of the recognition parameter according to Formula 11:

(11) $$Z_{i}=\{\begin{array}{cc} \frac{f_{i}}{L_{i}}\times H2_{i}\times (1-p_{i}) & f_{i}>L_{i}\\ \frac{f_{i}}{L_{i}}\times H1_{i}\times p_{i} & f_{i}\leq L_{i} \end{array}$$

where $Z_{i}$ represents the deviation of the amount of information of the recognition parameters of the test iris at their respective probabilities (greater or less than the average $L_{i}$). In the ideal state, $f_{i}$ and $L_{i}$ should be the same, but they will definitely be different. Therefore, the information offset is obtained by multiplying the ratio by the respective probability and information entropy. Formula 12 is used to calculate the entropy feature G, where G is the sum of the ratio of the information offset $Z_{i}$ to the category label $H1_{i}\times p_{i}+H2_{i}\times (1-p_{i})$ of each category. In the ideal state, $Z_{i}$ should be 0.5 times that of the following:

(12) $$G=\sum_{i=1}^{15}\left|\frac{2\times Z_{i}}{H1_{i}\times p_{i}+H2_{i}\times (1-p_{i})}\right|$$

Although the value of the entropy feature G in each category of the test iris is not the same, it might be relatively similar. Therefore, the entropy feature G is enlarged by the exponential function based on *e* to form the category label S. Based on statistical ideas, we summarize the expanded scope of the category labels in each category. Because this paper focuses on lightweight iris recognition, even if we apply the multi-range setting of category labels, different iris categories can also be well distinguished. In Formula 13, $\begin{array}{ccc} \left[\alpha 1_{i},\beta 1_{i}\right] & \cdots & \left[\alpha n_{i},\beta n_{i}\right] \end{array}$ represents different category label ranges (the interval between each interval range is set to $\lambda_{1},\ldots ,\lambda_{n}$; these values are not necessarily the same, and they are set based on the number of irises and the resulting distribution. The entropy feature G of the iris in this category will fall in the range after it is enlarged. For entropy features that fall within this range, we multiply them by 100 to expand and facilitate the calculation of matching values in the sigmoid function and enlarge the matching values of the same category.

(13) $$S=\{\begin{array}{ccccc} 100\times G & e^{G}\in & [\alpha 1_{i},\beta 1_{i}]\cap & \cdots & \cap [\alpha n_{i},\beta n_{i}]\\ G & e^{G}\notin & [\alpha 1_{i},\beta 1_{i}]\cap & \cdots & \cap [\alpha n_{i},\beta n_{i}] \end{array}\;HD=\frac{1}{1+e^{-S}}$$

After the output layer gets the certification result, the certification threshold is set to *L* (the specific value is set according to the condition of the iris, and the set value is greater than 0.9), and the values of *HD* and *L* are compared. If HD is greater than *L*, the image is considered to be of the same person.

The nature of the certification process is judged according to the degree of coincidence between the calculated entropy features and the distribution of category labels. $L_{i}$ and $p_{i}$ are the basis of all parameters; all parameters in formulas 1–5 are calculated according to these values. $L_{i}$ and $p_{i}$ are obtained from all the feature data extracted from the training iris, and the impact of $L_{i}$ and $p_{i}$ on certification are reflected in the category label range $\begin{array}{ccc} \left[\alpha 1_{i},\beta 1_{i}\right] & \cdots & \left[\alpha n_{i},\beta n_{i}\right] \end{array}$ of the final training data (enlarged area of entropy feature expansion of the same category). Therefore, during the test, if the entropy feature expansion value of the test iris is in the category label range, the input value of the sigmoid function is expanded to calculate the certification judgment result.

### 4.3. Feedback Learning Mechanism of Iris Certification

The feedback learning mechanism of the certification function is based on statistical ideas. With the increase of the number of test irises and the increase of recognition error conditions, it is determined whether to adjust and modify the function parameters. First, we accumulate the certification error conditions to determine whether the recognition parameters need to be modified. As the amount of data and the number of certification errors increase, we modify the parameters in certification functions 1–5 in turn according to the statistics of the data distribution.

Because of the causal effects of the parameters between the functions, the $L_{i}$ and $p_{i}$ of all the training irises are mainly adjusted, and then the range intervals $\lambda_{1},\ldots ,\lambda_{n}$ and $\begin{array}{ccc} \left[\alpha 1_{i},\beta 1_{i}\right] & \cdots & \left[\alpha n_{i},\beta n_{i}\right] \end{array}$ are modified as $L_{i}$ and $p_{i}$ change to achieve dynamic learning. Such measures enable the entropy features of the function and the range of the category labels to be dynamically adjusted as the number of iris increases, thereby achieving a current optimal effect.

The specific steps of the feedback learning mechanism are as follows:

1. The number of certification errors within a certain number of tests are counted and it is determined whether the parameters need to be adjusted based on the changes in the training data. This article assumes that the correct recognition rate is less than 95% or the number of training irises is increased by more than five times (where the new training iris contains the previous test iris); then, the certification model needs to be updated.
2. If it is determined that the update of the certification model is not required, the original set parameters and category label range for certification are used.
3. If it is determined that the certification model needs to be updated, new statistical adjustments are made to $L_{i}$ and $p_{i}$ based on all existing training data and according to the maximum satisfaction principle (choosing to satisfy the data distribution as much as possible). $L_{i}$ and $p_{i}$ are recalculated based on the recognition parameters of all training irises. The certification function group with formulas 1–5 is recalculated according to the new $L_{i}$ and $p_{i}$, according to the new entropy feature G, and the distribution of the expanded values, and this is used as a basis to count the new category label range and range interval.
4. Steps 1–3 are repeated, until a situation that cannot optimize the certification model appears (because of training data exhaustion, etc.).

## 5. Experiments and Analysis

**Experimental data:** All experiments in this paper use the JLU-4.0 (device in Figure 2a), JLU-6.0 (device in Figure 2c), and JLU-7.0 (device in Figure 2e) iris libraries [40]. The key indicators of the three acquisition sensors are shown in Table 1.

The JLU-6.0 iris library is a low-end acquisition device, while JLU-4.0 and JLU-7.0 are advanced devices. As of 2020, the JLU-6.0 original iris library contains more than 100 categories of irises, and each category contains more than 1000 unstable irises photographed in various states (including the standard morphology set). The JLU-4.0 original iris library contains more than 50 categories of iris, and each category has thousands of unstable irises photographed in various states (including the standard morphology set). The JLU-7.0 original iris library has more than 30 categories of iris, and each category has more than 1000 unsteady irises (including standard morphology sets) taken in various states. 

**Experimental setup:** In these experiments, the computing system included a dual-core 2.5 GHz CPU with 8 GB memory, and the operating system was Windows.

Section 5.1 gives the explanation of the structural meaning. Section 5.1.1: The relationship between iris features and iris requirements. From the quality evaluation process, it explains why iris quality has an impact on recognition results. Section 5.1.2: Reasonable structural design description. From the perspective of the overall design of the certification structure, it explains why this paper did not improve on other deep learning frameworks. Section 5.1.3: Structural properties and algorithm independence: From the feature expression process in the certification structure, the rationality of the structure design of this paper is indicated by replacing the algorithms in the recognition method and not using feature fusion. Section 5.1.4: Significance of certification function. From the perspective of the certification function design in the certification structure, it explains the design significance of the authentication function in this paper. Section 5.1.5: Reasonable setting of the feedback learning mechanism. From the perspective of the feedback mechanism, it explains the design significance of the feedback learning mechanism from two aspects: quality concept and design label.

Section 5.2 describes the performance of the algorithm. Section 5.2.1: Sensor heterogeneous versatility experiment: Explains the overall structure of the method proposed in this paper for heterogeneous certification. Section 5.2.2: Certification performance: Explains the experiment and the performance of single category certification. Section 5.2.3: Time operation experiment: It shows the time running of the process from confirming the collection to outputting the results in the one-to-one certification in this paper.

Section 5.3 contains a comprehensive experiment. Under the prerequisites of this paper, the processing structure of this paper is compared with the existing mechanical processing algorithms and the fixed combination of existing recognition algorithms, which illustrates the impact of iris processing and feature expression recognition algorithms on iris certification in the case of improper matching and the advantages of this method compared with other methods.

### 5.1. Explanation of Structural Meaning

#### 5.1.1. The Relationship Between Iris Features and Iris Requirements

**Experimental settings and indicators:** Several feature expression and recognition algorithms were listed. According to the requirements of feature expression and recognition algorithms for iris quality, the relevant iris concepts from the quality knowledge system illustrated in this paper and literature [41,42] were selected. One category was selected in JLU-6.0, and the iris was evaluated by certain evaluation indicators. The appropriate method to infer the iris images that meet the requirements (the number of iris images in each category in the initial iris library is 1000, the internal iris shape is different, and no quality evaluation is performed) was selected, and all qualified irises were determined to be training irises. Additionally, all of the irises were used as test irises to perform one-to-one certification tests (to identify the appearance of default overfitting and other situations). By exploring whether the iris that meets the requirements of the feature extraction algorithm is available, the rationality of the iris quality knowledge system designed in this paper can be explained.

The enumeration algorithms are as follows:

1. **Fusion method:** Iris recognition based on a feature weighted fusion method based on Haar and LBP in [17] and training feature weights through statistical learning;
2. **Secondary iris recognition:** Multicategory secondary iris recognition based on a BP neural network after noise reduction by principal component analysis, as used in [7]:
3. **DPSO-certification function:** A certification function optimization algorithm based on a decision particle swarm optimization algorithm and stable feature, as used in [16];
4. **Statistical cognitive learning:** A multistate iris multiclassification recognition method based on statistical cognitive learning, as used in [20];
5. **Multialgorithm parallel integration:** An unsteady state multialgorithm parallel integration decision recognition algorithm in [18];
6. **Capsule Deep Learning:** Iris recognition based on capsule deep learning in [13].

In the case of determining that the photographer is in a living state, according to the quality requirements of the iris images of each algorithm, the degree of the quality requirements of the iris indicators of each algorithm is shown in Table 2.

According to the design of the recognition algorithm structure and the nature of each indicator in the knowledge base, the evaluation knowledge selected by the six algorithms is as follows:

1. Fusion method: Clarity selects the secondary inspection method in literature [41]; the effective iris area detection uses the gray histogram distribution detection in literature [42]; the strabismus selects the eccentricity detection in literature [42]; and confirmation of the presence of eyes uses the eye concept cognition in the examples presented in this paper.
2. Secondary iris recognition: Clarity selects the secondary inspection method in literature [41]; effective iris area detection uses the method of detecting the appropriate iris area by using the block gray value variance in literature [41]; the strabismus selects the strabismus detection in literature [41]; and confirmation of the presence of eyes uses the eye concept cognition in the examples presented in this paper.
3. DPSO-certification function: Clarity selects the image blur detection in the example presented in this paper; the effective iris area detection uses and the presence of eyes selects the concept awareness of the eye in the example presented in this paper; and the strabismus selects the strabismus detection in literature [41];
4. Statistical cognitive learning: Clarity selects the image blur detection in the example presented in this paper; the effective iris area detection uses and the presence of eyes selects the concept awareness of the eye in the example presented in this paper; the strabismus uses the image direct vision detection in the example presented in this paper.
5. Multialgorithm parallel integration: Clarity selects the image blur detection in the example presented in this paper; the effective iris area detection uses and the presence of eyes selects the concept awareness of the eye in the example presented in this paper; the strabismus uses the image direct vision detection in the example presented in this paper.
6. Capsule deep learning: Clarity selects the sharpness test method used in literature [42]; effective iris area detection uses the method of detecting the appropriate iris area using the block gray value variance in literature [41]; the strabismus selects the strabismus detection used in literature [41]; confirmation of the presence of eyes uses the eye concept cognition in the examples presented in this paper.

All the indicators have met the qualification standards. Table 3 shows the qualifications and certifications of the six methods.

Table 2 and Table 3 show that different feature extraction methods have different quality requirements for irises, and after selecting appropriate quality conditions according to the quality requirements, a rigorous one-to-one is passed to the qualified iris. The certification test found that most of the iris can be used, and this test proves that the exclusive setting of the quality evaluation conditions for the feature expression and recognition algorithm is reasonable.

Additionally, the sample knowledge base and other quality evaluation standards are used in this experiment. According to the quality requirements of different situations and different methods, different types of quality knowledge are selected for quality reasoning, thus showing that the iris quality evaluation standard is not a fixed scale. The standard should be expanded and revised as external knowledge increases. Therefore, the feedback dynamic learning mechanism designed in this paper is reasonable and meaningful. The mechanism can dynamically modify and expand the knowledge base according to the changes in iris quality requirements and prevent the omission of identifiable irises because of fixed indicators.

#### 5.1.2. Reasonable Certification Structural Design Description

**Experimental setup:** In this section of the experiment, the CNN architecture of this paper (CNN-special certification function) and a variety of deep learning architectures are used to perform a pair of category certification experiments; here, it is explained why the CNN framework in this paper was used and the prerequisites and innovation of this paper are identified.

For iris localization and normalization, we adopted a method in which the normalized size of the iris recognition area is 180 × 32 dimensions, and the quality confirmation had no effect on iris certification. The test iris and training iris in the three iris libraries were the same. The number of iris categories and the number of irises in a single category are shown in Table 4. The training iris and testing iris met the prerequisites of this paper, excluding extreme cases of interference. For the multi-source features in the experiment, Gaussian filtering (smoothed) and the equalizing histogram (highlighted) were used as image-processing algorithms. The convolutional kernel of the image processing is the example convolutional kernel of this paper.

The comparative deep learning architectures are as follows:

1. Faster R-CNN Inception Resnet V2(FRIR-V2) in [43]; 2. VGG-Net in [44]; 3. DeepIrisNet-A in [15]; 4. DeepIris in [14]; 5. DeepIrisNet in [24]; 6. Alex-Net in [45]; 7. ResNet in [46]; 8. Inception-v3 in [47]; 9. CNN with self-learned features generated (CNN-self-learned) in [48]; 10. Fully convolutional network (FCN) in [49]; 11. DBN-RVLR-NN in [50].

The certification statuses of all 10 deep learning architecture methods are shown in Table 5.

As one of the most popular research tools, deep learning is of great significance in pattern recognition. There are also many deep learning architectures that have CNN as their main object and have good results. However, in terms of iris recognition, the time for the application of deep learning in iris recognition is relatively short, and the existing deep learning architecture has the following problems:

1. The existing structural framework is designed to process a certain type of data, and the design purpose of the framework is not necessarily aimed at iris recognition. Therefore, when the existing frame is used for iris recognition, inputting iris data directly into the frame may not achieve a good recognition effect;
2. At present, there is no detailed definition of iris features. The expression of iris features is usually determined after the iris is converted from an image into a digital form, which has certain requirements for the internal feature transformation of the deep learning architecture. When faced with a multi-sensor iris whose acquisition status is unpredictable, there will be a large number of frameworks that cannot adapt to this kind of situation, which will lead to a decline in the accuracy of identifying irises from different acquisition sensors;
3. The architecture of deep learning has high requirements for the classification of data volume and data situation. In addition, for a certain deep learning framework, the computing power is highly related to computing equipment, making it difficult for many low-level devices to use deep learning architectures for calculations.

In actual research on iris recognition, there will not be much iris data—only lightweight data will be present. However, even for the same person’s iris, because the collector is not guided and the types of the collection sensors are different, the same deep learning architecture does not learn enough about the iris category, with different effects on different sensors. Expensive equipment and cumbersome training processes will hinder the promotion of iris recognition technology, and thus it is necessary to design a simple certification architecture for such situations.

In the analysis based on the data in Table 5, as far as the commonly used deep learning architectures are concerned, this paper is an improvement on the basic CNN architecture, but this is the most suitable scheme designed under the prerequisites of this paper. The VGG-Net architecture, Alex-Net architecture, ResNet architecture, and Inception-v3 architecture are not designed specifically for iris recognition. Therefore, the data need to be converted into a form that can be recognized by the architecture, and need to be classified according to different collection statuses. In addition, it is possible to improve the accuracy of recognition only when a large amount of data is used for training. The prerequisites of this article do not match, making the recognition accuracy lower than this paper.

DeepIrisNet-A architecture and DeepIris architecture are a specialized iris recognition framework, which includes eight convolutional layers (each has a small standardized layer after each layer), four pooling layers, three fully connected layers, and two drop-out layer. These architectures can achieve good results in existing public iris libraries. However, the prerequisite for this article is a small sample multi-state training set. The number of iris samples and classifications cannot meet the needs of large-scale iris recognition model training, so the recognition accuracy is low. The FCN architecture and the DBN-RVLR-NN architecture are improvements to the existing architecture. This type of architecture improves the way of learning and feature extraction, thereby improving the recognition effect. However, they have certain requirements for images, which make feature learning of multi-state images easy to miss, so the recognition accuracies are lower than this paper.

Using the same iris recognition model for different irises for certification, the migration learning iris recognition framework DeepIrisNet is used for lightweight training samples to solve the situation in which the data set is not large enough. The improvement of the recognition structure through transfer learning can effectively train with lightweight training samples and improve the certification accuracy. The Faster R-CNN Inception Resnet V2 model architecture is an advanced convolutional neural network model that allows training with lightweight data, which can reduce the difficulty of training. The reason why the certification effect is not as good as in the structure of this paper is that the prerequisite of this paper is an unpredictable multi-state iris. Compared with the traditional public iris library, the experimental iris shape is changeable, and it is difficult to classify the situation. Training can only be performed on mixed data, thus reducing the training effect.

Under the prerequisites of this paper, when we have only a small amount of data that mixes irises from different acquisition states, in order to ensure that the same structure can play a role for different acquisition sensors, the structure of this article focuses on the design of the authentication function later. Under the role of a convolution kernel that highlights the edge of the iris texture as much as possible, considering the amount of data and the few categories to be distinguished, according to the situation of data distribution, a non-linear function is used to expand the entropy features, making sure there is no intersection between different categories, and making the certification feature tags of the same class unique. Therefore, under the prerequisites of this paper, although this paper only uses an improved typical CNN structure, this structure can still have a better effect than other deep learning architectures.

#### 5.1.3. Structural Properties and Algorithm Independence

**Experimental setup in this section:** The knowledge base of the quality evaluation concept system itself is carried out in a mechanism that can be expanded and modified. Therefore, the experiments in this section are to verify the algorithm independence of the recognition model structure. The algorithm in this paper is replaced with the multi-source image-processing algorithm to verify the relationship between the algorithm structure design and the specific algorithm. Part 1 is feature extraction using only the original image, without feature smoothing and highlighting. Part 2 is the replacement of the original Gaussian filtering and equalization histogram with median filtering [51] (smoothed) and the Laplace operator [52] (highlighted). The reason for choosing these two filtering operations is because it is also a common smoothing and highlighted filtering image. Using these two components of the experiments, the design significance of the multi-source feature fusion of the method in this paper is explained, and the mechanism is independent of the specific algorithm. The convolutional kernel of the image processing is the example convolutional kernel of this paper.

In this section of the experiment, the same iris category as the certification experiment is selected, with each category having 2000 initial training irises (the quality evaluation indicator is the evaluation indicator exemplified in this paper). The test iris and training iris of each category iris photographed in the same state in each category experiment meet the prerequisites of this paper and exclude extreme interference. The comparative test irises are all irises inside the experimental iris library, and the number of test irises is 1000 (after passing the quality assessment of this paper, this is qualified and will not affect the certification). The experimental results in the first-part and second-part experiments are shown in Table 6.

It can be observed from Table 6, after the algorithm is changed, the overall certification effect does not appear to fluctuate greatly; the original image processing is used, and the certification effect is greatly reduced. Therefore, it can be concluded that the setting of the multi-source feature fusion mechanism in the method in this paper can improve the recognition effect, but the recognition effect has little correlation with the specific image-processing algorithm. The multi-source processing image mechanism of the method in this paper is designed to achieve the purpose of feature fusion by smoothing processing, highlighting, texture processing, and the numerical averaging of the original image. The reason for this design is that in the case of an unsteady iris, it is impossible to determine the appearance status of the iris feature points. Therefore, once the training iris is set in a fixed state, it is easy to increase the difficulty of matching the test iris with the template iris (e.g., the training iris is a defocused image, and the texture is blurred; however, the test iris is a clear image, and the texture is prominent, which causes a large difference between the values after convolution processing). Although training via a large amount of data can make up for this shortcoming, due to the unpredictable state, it is difficult to fully obtain all of the cases when the data set is incomplete, and it is easy to encounter different categories of edge interactions.

Therefore, the method in this paper adopts a neutralization method. By averaging the values of the original image, the smooth image, and the texture highlighted image, the values after convolutional processing are concentrated to a certain extent so as to deal with the entropy of the subsequent recognition function. The clustering of features creates conditions to avoid the adverse effects of highly scattered data on clustering. This mode does not place overly high requirements on the image-processing algorithm itself, and therefore, even if the image-processing algorithm is replaced, the overall recognition effect is not affected.

#### 5.1.4. Significance of Certification Function

**Experimental setup in this section:** One training iris in the three iris libraries waws taken as an example. The changes of each parameter in 100 training images and 500 training images were compared, and the rule of parameter selection in this paper was explored. $L_{i}$ and $p_{i}$ of 15 recognition parameters of 500 training images and 100 training images are shown in Table 7. The information entropy $H1_{i}$ and $H2_{i}$ of 15 recognition parameters of 500 training images and 100 training images are shown in Table 8. The 15 values of the category labels of the three iris libraries are shown in Table 9. The recognition parameters of the example training iris are shown in Table 10. The information offset $Z_{i}$ of the example training iris is shown in Table 11. The entropy feature G and enlarging value $e^{G}$ are shown in Table 12. 

According to the changes of all the parameters of the training iris in the example, it can be seen from the data changes in Table 7, Table 8, Table 9, Table 10, Table 11 and Table 12 that the entropy feature expansion values finally obtained by the same recognition parameter in different numbers of training iris are different. Because the iris defined in this article is a constrained iris, which also eliminates extreme collection situations, the quality of the iris itself is considered to be a digital representation of features. Therefore, even with a different number of training irises, its own distribution will allow entropy feature values to cluster into certain regions. 

The method used in this paper to distinguish different features analyzes the distribution of the entropy feature expansion values of all the training irises. Under the condition that the data can form a certain degree of clustering, select the appropriate category interval to mark a fixed category. Because in the calculation based on different category labels, there must be disjointed areas for different categories. For example, in the process of performing the same-category certification of category A, the entropy feature expansion value based on the category label A can only fall in the category interval B. An iris that is not in category A can also be excluded if the entropy feature expansion value is in the category interval B, because the basis of the calculation is not the category label of category A. Through this mode, different categories can be effectively distinguished in lightweight same-category certification, and the accuracy of one-to-one certification in the same category is guaranteed.

The data difference within the same category is small, and closer clustering can be achieved. However, due to the limitation of the number of recognition parameters, the difference in entropy features is not obvious in certain cases. Therefore, the exponential function with the base *e* is expanded to expand the value of the entropy features, thus greatly expanding the differentiation among the categories of irises and the performance of one-to-one certification. The entropy features of the iris in the same category are mostly concentrated in a certain region. After expansion, due to the multi-state iris, the enlarged iris image is concentrated in different regions. Even if the entropy features are relatively close, they can be expanded to a large gap. Because the prerequisites for this paper require running the tester to retake the iris image, this paper uses the multi-range method. Using reasonable range intervals, the enlarged data of different categories of iris after the same category label can be effectively distinguished and will not coincide. This result can greatly improve the accuracy of certification.

#### 5.1.5. Reasonable Setting of the Feedback Learning Mechanism

##### Reasonable setting of quality concept

**Experimental setup:** In this section of the experiment, the quality of iris images in one category of the three iris libraries is evaluated (1000 images per iris library, without prior classification, and the iris normalization dimension is 180 × 32). Using the multi-indicator mechanical determination method in [41], three types of indicators were selected: sharpness, squint, and effective iris area. First, one indicator was used for quality evaluation, and then the three indicators were used for quality evaluation. The qualified iris images were converted into 512-bit binary feature codes by Haar wavelet, and the iris of the same category in the same iris library were matched with each other by Hamming distance, and the problem of mechanical determination index was analyzed through the recognition result of the same category matching.

The results of the quality evaluation and recognition of the four cases in the experiments in this section are shown in Table 13, where:

Case 1: represents only using the indicator of sharpness; Case 2: represents only using the indicator of squint; Case 3: represents only using the indicator of effective iris area; Case 4: represents the situation where the three indicators are used together.

According to the previous experiment, it can be known that the steady-state iris has an effect on the expression and recognition of features, so the accuracy value is not important. The observation index of this experiment is the change of accuracy in different situations. When using a single indicator for evaluation, although there are more qualified images than multiple indicators, the accuracy rate is not as high as the multiple indicators. Because of the unsteady state iris, there are many situations that affect the quality of the iris, which are the result of a variety of factors. It is difficult to judge the quality completely with a single indicator or mechanical indicator, and because the matching degree of image quality and recognition algorithm is not high, the selected “qualified image” may not be suitable for the feature extraction algorithm. These two points cause the final accuracy rate to not be high. Therefore, this paper focuses on feature extraction and recognition methods, takes cognition as the lead, and formulates a quality evaluation mechanism that can be modified and expanded according to the external environment. The image quality is conceptualized to make it adapt to different algorithms as much as possible, and the feedback mechanism allows it to be flexibly adjusted according to specific circumstances. This method is more flexible and more reasonable, and can effectively solve the problem of reverse modification of concepts in the process of quality evaluation.

The significance of the setting of the feedback mechanism of the certification module

**Figure 26:**
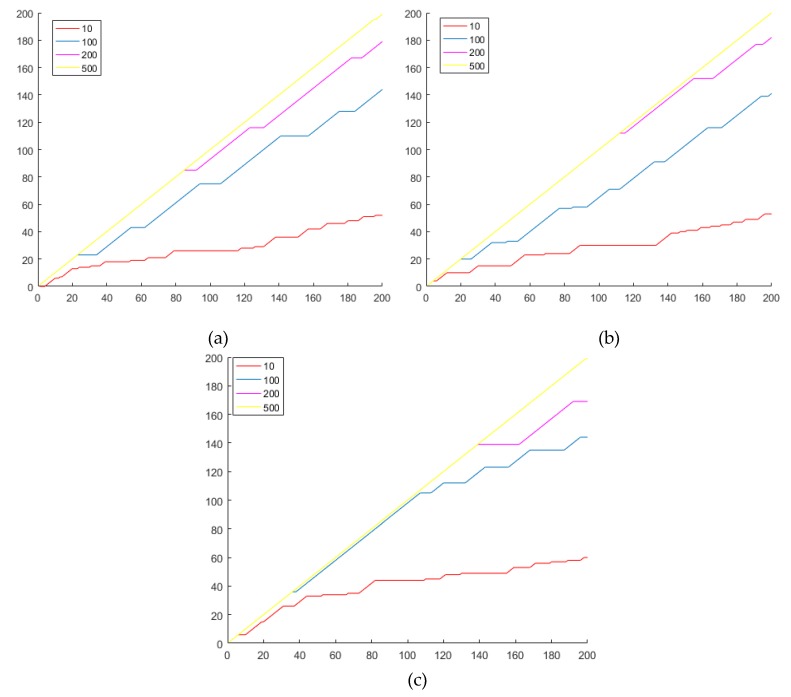
The growth trend of 200 test irises, (**a**) 4.0, (**b**) 6.0, (**c**) 7.0.

**Experimental setup in this section:** After ensuring that the quality of the iris did not affect the recognition results, each iris library selected one category and used the certification function designed in this paper for iris certification. The convolutional kernel of the image processing was the example convolutional kernel of this paper. The growth trend of 200 test irises (experimental test images and training images) without a feedback mechanism when the training irises consisted of 10, 100, 200, and 500 images in the three iris libraries is presented in Figure 26. 

In Figure 26, the abscissa represents the serial number of the test image, and the ordinate represents the number of irises recognized correctly. The feedback learning mechanism was used to dynamically modify the certification function (the average $L_{i}$ and probability values $p_{i}$ of the recognition parameters and the range of the category interval). Each of the three iris libraries selected a certain category of iris.

Although the design of the certification function guaranteed the accuracy of the iris certification results, it can be observed from Figure 26 that, when the number of trained irises was small and constant, if the number of tested irises was much larger than the number of trained irises, the accuracy rate was expected to decrease. Because the number of training irises was insufficient to make the selected category label range insufficient, the information entropy value could not be guaranteed to be always available. Therefore, it was necessary to increase the training data in time to adjust the recognition model, but the user needed to know when the recognition model needed to be adjusted to avoid unnecessary adjustment. Thus, it was necessary to design a feedback learning mechanism.

Table 14 shows examples of different amounts of training, numbers of iris recognitions, and updated examples of the certification situation. This paper assumes that if the correct recognition rate is less than 95% or if the number of trained irises is increased by more than five times (where the new training iris contains the previous test iris), then the model needs to be updated. After judging that the certification model needs to be updated, the numerical changes of the parameters of the iris category range under different numbers of training iris are shown in Table 15 (the range of iris categories is chosen as an example because the changes in the average and probability values can ultimately be reflected by the range of iris categories).

In the example of the feedback learning mechanism shown in Table 14, it can be observed that the number of irises trained affects the accuracy of the large-scale test iris, and thus dynamic adjustment is necessary. The number of training irises should ensure the correct recognition rate of the test iris within a certain number, and the designed feedback learning mechanism can prompt the user to increase the training iris data and adjust the new recognition model (adjusting the average number and concept, adding new category interval ranges) such that the recognition model always maintains a high accuracy rate and makes up for the problem of the dynamic adjustment of the convolutional neural network structure under the training of lightweight data.

Because $L_{i}$ and $p_{i}$ are adjusted according to all the training irises that they have, as the amount of training iris data increases, these two values will constantly change; then, they will reflect the entropy feature and the entropy feature expansion value. It can be seen from Table 15 that as the number of training irises increases, the category label distribution area (the area in which the entropy feature expansion values are gathered) is constantly increasing. Because there will always be new clustering areas, the parameters of the certification function in this paper need to be continuously changed with the number of irises. The setting of any parameter is determined based on the number of existing irises. In the case of a multi-state iris, it is difficult to simply cluster all images into a small area with simple entropy features. The expansion of non-linear functions makes the distinction between different types of iris greatly expand, which will prevent the clustered areas of different types of iris from overlapping, which will improve the accuracy of certification.

### 5.2. Certification Method Performance Experiment

#### 5.2.1. Sensor Heterogeneous Versatility Experiment

**Experimental settings and indicators:** In this section of the experiment, each of the three iris libraries uses five categories as the verification data, and the parameters of each category are trained by 500 training images qualified by the quality evaluation system (without new feedback learning adjustments). In one-to-one certification for each category for separate collection tests, each category directly collects one iris for individual matching. The tester’s iris was collected without the guidance of posture correction and normal shooting. One-to-one certification of each category of the same iris library was selected for comparison. For the multi-source features in the experiment, Gaussian filtering (smoothed) and the equalizing histogram (highlighted) were used as the image-processing algorithms. The judgment indicators for the results of this experiment are listed as follows:

1. The number of iris acquisitions when each category of each iris library reaches 100 accurate certifications (which is divided into: the number of repeated acquisitions due to quality evaluation problems, and the number of repeated acquisitions caused by system false rejection (no problem in quality detection)).
2. The number of errors identified when each category of each iris library reaches 100 times was accurately identified.

The results of the heterogeneous universality experiment are shown in Table 16.

It can be seen from Table 16 that in the process of iris certification using the same set of structures in multiple iris libraries, the iris’ false acceptance rate is extremely low, and the occurrence of redundant shooting is basically based on the quality of the non-compliance and false rejection. The collector is deemed not to be in the template iris category. This is because in the case of allowing repeated shooting, the quality evaluation is based on the actual shooting conditions of each iris, the subjective feeling concept is digitally expressed in a suitable way, and there is no fixed indicators used. This paper uses the example of quality evaluation indicators to consider the differences between different iris libraries when setting, based on a large number of non-thresholding processes, so the three libraries use the same indicator. Each parameter in the iris recognition function is based on the training iris, and the different people’s features are greatly diluted by the entropy feature expansion effect, thereby improving the possibility of parameter value cross over between different people by the range interval. To improve the model the samples must be differentiated, the further dilution range of the feature value of the certification function must be narrowed, the matching value of the same category as much as possible must be increased, and the matching value of different categories must be reduced. Therefore, in the formal test, because the quality evaluation conditions are extensive and the range interval compresses the range of some parameters, the user needs to take a new image, but the overall number is not large and is within the acceptable range. In addition, although there are very few cases of incorrect certification, the feedback learning mechanism in this paper can handle new situations, adjust the problems in the certification structure in time, and improve the certification accuracy.

From these, it can be concluded that the overall recognition structure designed in this paper can effectively operate in different iris banks, has good heterogeneity and universality, can effectively operate in multiple iris libraries, and the overall difference is not high.

#### 5.2.2. Certification Performance Experiment

**Experimental indicators:** The multi-source features in the certification performance experiments used Gaussian filtering (smoothing) and the equalization histogram (prominence) as multi-source image-processing algorithms. The evaluation indicators included the correct recognition rate (CRR) and ROC space (curve) (including false positive rate (FPR), true positive rate (TPR), and area under curve (AUC)) [53]. The ROC space (curve) defines the false positive rate (FPR) as the x-axis and the true positive rate (TPR) as the y-axis. Because the prerequisite requirement for this article was to guarantee accuracy and allow users to re-shoot, the case of false rejection was also considered true. 

**Figure 27:**
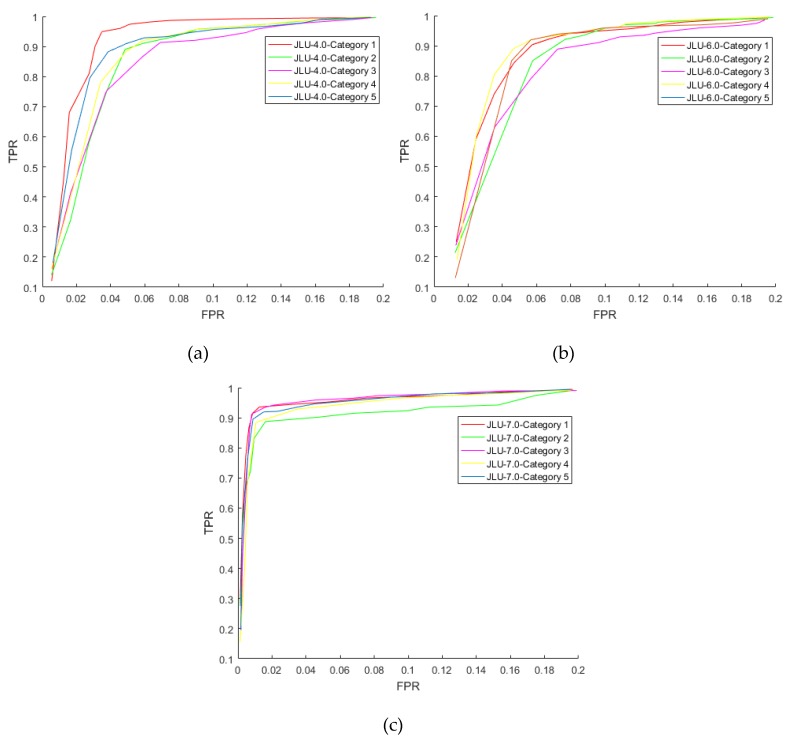
ROC curves of one-to-one certification of each category of the three iris libraries, (**a**) 4.0, (**b**) 6.0, (**c**) 7.0.

**Experimental setup in this section:** In this section, each of the three iris libraries was selected from five categories for experimentation, and the parameters of each category were trained from 2000 training images which qualified for quality evaluation (without new feedback learning adjustments; iris quality does not affect certification). The convolutional kernel of the image processing is the example convolutional kernel of this paper. During the test, the same category and different categories were used in one-to-one certification (originating from the same iris library, and the test iris and training irises are different). We observed the experimental data for analysis. Table 17 shows the recognition times of each category in different iris libraries. The threshold range of the ROC curve in this experiment was [0.5, 1], and the interval was 0.01. The ROC curves for single-category recognition of each category in the three iris libraries are shown in Figure 27.

From Figure 27, under the condition that the number of training irises is guaranteed, although the certification effect of each category of different iris libraries is different, the overall area under the curve (AUC) is significantly larger than 0.5, indicating that the accuracy of each single-category certification for each category is high, and the method has good heterogeneity. If the amount of data is sufficient, the method has good predictive value.

According to analysis of the results in Table 17 and Figure 17, due to the unpredictability of the multi-state iris acquired by different acquisition sensors, the feature appearance between different iris images might be notably different, which is reflected probabilistically in the amplitude value and distribution after edge detection based on the limitation of the scale and situation classification of the iris data set, which makes it impossible to use a large-scale deep learning architecture. The certification function of the method in this paper is based on the current iris data when performing one-to-one certification. The same category labels and recognition parameters are clustered in the same category, and the parameters are differentiated according to the probability of different amplitude values. Features are extracted in the form of information entropy to find the connection between the images of the same category to the extent possible. Iris features of the same category can be grouped together such that features of different categories cannot be aggregated. In expanding and diluting the non-linear function, the clusters of the same category in the multi-state iris are strengthened by setting multiple label ranges. Compared with the mechanical single threshold judgment, the recognition range is larger, and the structure setting is more flexible.

#### 5.2.3. Time Operation Experiment

**Experimental indicators:** Under the prerequisites of this paper, the images were selected from JLU-4.0, JLU-6.0, and JLU-7.0 iris libraries to constitute the experimental iris library. The classification thresholds of the three algorithms in the recognition model are trained by 1000 training iris images (50 categories are selected from each iris library and 20 images are from each category). The test iris used a series of 10 additional images in the same category (1000 in each iris library). The test irises have not passed the quality judgment. All irises will pass all the iris recognition processes and test the certification time of all processes.

The test irises for continuous certification time (milliseconds (ms)) and the certification situation are shown in Table 18.

It can be seen from Table 18 that after 1000 consecutive one-to-one certification experiment in the same category, the running times of the entire iris certification process in different iris libraries are within 6000–6500 ms. In the case of ensuring that the front-end preprocessing is completed, the certification time of a single image was 6–7 ms, which met the actual work requirements.

### 5.3. Comprehensive Experiment

Experimental setup: In this section of the experiment, the overall process structure of this article is compared with a combination of various existing quality evaluation and recognition methods (localization and normalization adopt this method, the normalized size of the iris recognition area is 180 × 32 dimensions). The certification algorithms in the three iris libraries were trained using the qualified images detected by their respective quality evaluation algorithms (the training iris is not classified by state, and is mixed data). The test iris was the initial photographed iris without the quality evaluation algorithm. The number of iris categories and the number of iris in a single category are shown in Table 19. 

Training irises and testing irises met the prerequisites of this paper and exclude extreme situation interference. Training irises and testing irises were completely different. During the experiment, quality evaluation was first performed, recognizable irises were found by the quality evaluation method for iris one-to-one certification in the same category, and the identified irises were matched with the identified unrecognizable irises through the recognition algorithm for one-to-one certification in the same category, and the corresponding proportion to analyze the experimental results was calculated. For the multi-source features in the experiment, Gaussian filtering (smoothed) and the equalizing histogram (highlighted) were used as image-processing algorithms.

**The experimental evaluation indicators in this section include:** the number of recognizable irises considered by the quality evaluation method, the number of correctly identified recognizable irises and the correct certification rate (the number of correctly identified of recognizable irises/the number of recognizable irises considered by quality evaluation method), the number of correctly identified of unrecognizable irises and the correct certification rate (the number of correctly identified of unrecognizable irises/the number of unrecognizable iris esconsidered by quality evaluation method).

The overall certification structure (Case 0) in this paper is compared with the following 20 algorithm combinations:

**Mechanical quality evaluation indicator + traditional iris recognition algorithm:**

1. Case 1: Qualified iris evaluation indicators (biological detection, sharpness, effective area, strabismus, and transboundary) in [42] and iris recognition with the image processed by the log operator based on Gabor filter optimization and Hamming distance in [6].
2. Case 2: Qualified iris evaluation indicators (biological detection, sharpness, effective area, strabismus, and transboundary) in [42] and iris recognition based on feature weighted fusion in [17], which train feature weights through statistical learning.
3. Case 3: Inference engine system (sharpness, effective area, strabismus, and transboundary) of quality qualified iris evaluation indicators in [41] and a certification function optimization algorithm based on the decision particle swarm optimization algorithm and stable features in [16].

**Mechanical quality evaluation indicator + deep learning framework recognition algorithm:**

4. Case 4: Qualified iris evaluation indicators (biological detection, sharpness, effective area, strabismus, and transboundary) in [42] and iris recognition and prediction based on multi-view learning classifiers in [54].
5. Case 5: Inference engine system (sharpness, effective area, strabismus, and transboundary) of quality qualified iris evaluation indicators in [41] and concept cognition based on the deep learning neural network in [11].
6. Case 6: Inference engine system (sharpness, effective area, strabismus, and transboundary) of quality qualified iris evaluation indicators in [41] and a unsteady iris one-to-one certification method based on statistical cognitive learning in [20].

**Quality evaluation fuzzy inference + traditional iris recognition algorithm:**

7. Case 7: Quality evaluation fuzzy reasoning system (example indicators in this paper) and iris feature representation based on the fractal coding method in [55].
8. Case 8: Quality evaluation fuzzy reasoning system (example indicators in this paper) and the histogram of oriented gradients (HOG) is used to extract the iris features and certification by support vector machine (SVM) in [56].
9. Case 9: Quality evaluation fuzzy reasoning system (example indicators in this paper) and feature extraction based on the scale-invariant feature transform (SIFT) in [57] and recognition based on SVM.

**Quality evaluation fuzzy inference +deep learning framework recognition algorithm:**

10. Case 10: Quality evaluation fuzzy reasoning system (example indicators in this paper) and iris recognition based on the cognitive internet of things (CIoT) identified by multi-algorithm methods in [58]
11. Case 11: Quality evaluation fuzzy inference (example indicators in this paper) and the iris recognition method based on the iris-specific Mask R-CNN in [12].
12. Case 12: Quality evaluation fuzzy inference (example indicators in this paper) and an iris recognition method based on error correction codes and convolutional neural networks in [22].

In the comprehensive experiment, the number of qualified irises in the three iris libraries developed in three different ways are shown in Table 20. The recognition results of all 12 cases in the three libraries are shown in Table 21.

From the experimental results in Table 20 and Table 21, it can be seen that the experimental results of the three iris libraries are relatively consistent. The study of the overall structure of the iris has a great impact on the certification of the iris. The algorithm does not match and the certification accuracy is low. An iris considered as unrecognizable may not necessarily be used in this certification algorithm. The overall structure of this paper has the highest certification accuracy for the detection of unsteady irises, and the lowest certification accuracy for the iris considered as unrecognizable.

Analysis of the experimental results in Table 20: The quality evaluation method of the mechanical threshold indicator for the purpose of detecting qualified images depends on the form of the template iris, and the process was intended to find an iris image similar to the template iris. Compared with the fuzzy system dominated by the unqualified knowledge system in this paper, the flexibility is insufficient. Therefore, the number of recognizable irises considered by the quality evaluation method in this paper is large. Because the unrecognizable irises considered by the system in this paper example is selected based on the poor quality knowledge concept in the unsteady irises, it is rare that the unrecognizable image can still get the correct result when it is recognized. However, the other two comparison methods are based on the eligibility criteria for judgment, so there may be cases where the recognizable irises (the irises matching the feature extraction certification algorithm) are mistakenly eliminated. The fuzzy system in this paper can be adjusted through the feedback dynamic learning mechanism according to external conditions and other methods, so the overall structure can be more flexible.

In summary, we can conclude that different iris feature extraction and recognition methods have different requirements for iris quality, so special quality requirements need to be designed according to different feature extraction and recognition methods. The certification architecture of this paper considers the connection relationship among different links, which can avoid the shortcomings of low adaptation among different links caused by the division of the whole process machinery, and solve the problem of correlation between each link. At the same time, it pays attention to the universality of the quality concept setting process and the certification structure, so that it is independent of equipment and environment. In addition, knowledge cognition is the main factor, and the concept of each component is established to prevent the problem of insufficient learning that restricts the different states of the unsteady iris due to the fixed threshold. Aimed at the lack of training data sets and training data classification, the features of the iris are expressed through multiple sources, and the existing single deep learning architecture model is improved. A new certification function was designed to improve the degree of discrimination between different categories, thereby improving the certification accuracy of one-to-one certification of the lightweight category of the certification module and the capability of heterogeneous iris certification.

## 6. Conclusions

Aiming at the unsteady iris caused by different devices and environments, this paper proposes a lightweight heterogeneous iris one-to-one certification overall process with universal sensors based on a quality evaluation fuzzy inference and a multi-feature fusion lightweight neural network. The focus is to resolve the issue that the traditional processing method of single-source irises in unsteady state iris is ineffective, and the correlation of each link caused by the mechanical segmentation of the iris certification process is insufficient. The existing iris data set size and situation classification constraints means that it is difficult to meet the requirements of learning methods in a single deep learning framework. The results of different iris libraries prove that the design of each part of the method is reasonable and meaningful. The accuracy in different iris libraries is maintained at a high level and the recognition range is wide. Through the feedback learning mechanism, experts dynamically adjust the model according to the results.

This paper mainly focuses on recognition forward flow process. For the reverse optimization process, only the relevant mechanism is designed. Making the mechanism more automated and letting the system perform unsupervised learning and multi-categories recognition are the next areas of research focus.

## Figures and Tables

**Table 1:** The key indicators of the three acquisition sensors.

	JLU-4.0	JLU-6.0	JLU-7.0
Pixel	5,000,000	2,000,000	300,000
Collection distance	150–200 mm	100–150 mm	150–200 mm
Resolution	640 × 480	640 × 480	640 × 480
Light	Infrared	Infrared	Non-infrared
Color	Color	Grayscale	Grayscale

**Table 2:** The degree of quality requirements of the iris indicators of each algorithm.

Method	Clarity (Degree of Light Effect)	Effective Iris Area	Strabismus	Confirm the Presence of Eyes
Fusion method	high	high	high	yes
Secondary iris recognition	high	medium	medium	yes
DPSO-certification function	low	low	medium	yes
Statistical cognitive learning	low	low	low	yes
Multialgorithm parallel integration	low	low	low	yes
Capsule deep learning	medium	medium	medium	yes

**Table 3:** The qualifications and certifications of the six methods.

	Method	Consider Qualified Number	Correct Certifications of Consider Qualified Number
JLU-4.0	Fusion method	156	155
	Secondary iris recognition	235	230
	DPSO-certification function	435	434
	Statistical cognitive learning	943	943
	Multialgorithm parallel integration	954	952
	Capsule deep learning	513	511
JLU-6.0	Fusion method	179	175
	Secondary iris recognition	258	257
	DPSO-certification function	474	473
	Statistical cognitive learning	963	958
	Multialgorithm parallel integration	974	973
	Capsule deep learning	526	523
JLU-7.0	Fusion method	111	110
	Secondary iris recognition	210	210
	DPSO-certification function	453	453
	Statistical cognitive learning	1007	1003
	Multialgorithm parallel integration	987	985
	Capsule deep learning	546	545

**Table 4:** The number of iris categories and the number of single-category irises.

Category Number	Image Number in Each Category	Total	Match Number in the Same Category	Match Number in Different Category	Total
50	50	2500	2500	7500	10,000

**Table 5:** The certification statuses of all 12 deep learning architecture methods.

	JLU-4.0		JLU-6.0		JLU-7.0	
Method	Correctly Certification	CRR	Correctly Certification	CRR	Correctly Certification	CRR
CNN-special certification function	9997	99.97%	10,000	100%	9998	99.98%
FRIR-V2	9135	91.35%	8421	84.21%	8754	87.54%
VGG-Net	7369	73.69%	8123	81.23%	7869	78.69%
DeepIrisNet-A	7869	78.69%	7498	74.98%	8096	80.96%
DeepIris	8456	84.56%	8569	85.69%	8375	83.75%
DeepIrisNet	9236	92.36%	9347	93.47%	9147	91.47%
Alex-Net	6789	67.89%	5698	56.98%	6845	68.45%
ResNet	7125	71.25%	7236	72.36%	7523	75.23%
Inception-v3	7698	76.98%	7748	77.48%	7496	74.96%
CNN-self-learned	8963	89.63%	8742	87.42%	8541	85.41%
FCN	8546	85.46%	8325	83.25%	8698	86.98%
DBN-RVLR-NN	7968	79.68%	7762	77.62%	8023	80.23%

**Table 6:** The number of comparison times and recognition situation.

		Number of Correct Certification	Correct Certification Rate
JLU-4.0	Original structure	998	99.8%
	Unprocessed structure	875	87.5%
	Structure of replacement algorithm	998	99.8%
JLU-6.0	Original structure	1000	100%
	Unprocessed structure	869	86.9%
	Structure of replacement algorithm	997	99.7%
JLU-7.0	Original structure	999	99.9%
	Unprocessed structure	892	89.2%
	Structure of replacement algorithm	1000	100%

**Table 7:** $L_{i}$ And $p_{i}$ of 15 recognition parameters of 500 training images and 100 training images.

The Number of Training Iris Is 500					The Number of Training Iris Is 100				
No.		JLU-4.0	JLU-6.0	JLU-7.0	No.		JLU-4.0	JLU-6.0	JLU-7.0
1	$L_{i}$	41.8608	15.7176	24.374	1	$L_{i}$	13.1152	18.8164	25.5812
	$p_{i}$	0.6	0.58	0.58		$p_{i}$	0.6	0.48	0.56
2	$L_{i}$	38.2424	25.0296	67.3079	2	$L_{i}$	56.6001	45.5503	54.137
	$p_{i}$	0.4	0.48	0.48		$p_{i}$	0.4	0.48	0.5
3	$L_{i}$	15.127	16.8023	49.4673	3	$L_{i}$	27.1576	27.717	35.3843
	$p_{i}$	0.4	0.58	0.5		$p_{i}$	0.6	0.58	0.52
4	$L_{i}$	54.5455	19.2285	32.8769	4	$L_{i}$	27.3758	27.0261	16.1855
	$p_{i}$	0.6	0.54	0.48		$p_{i}$	0.6	0.54	0.48
5	$L_{i}$	25.5758	24.157	54.8345	5	$L_{i}$	36.2848	40.5636	47.3764
	$p_{i}$	0.4	0.48	0.42		$p_{i}$	0.4	0.56	0.48
6	$L_{i}$	26.0909	20.8958	55.0618	6	$L_{i}$	40.3152	33.6909	42.1176
	$p_{i}$	0.4	0.58	0.46		$p_{i}$	0.4	0.46	0.6
7	$L_{i}$	38.8545	13.9576	25.9436	7	$L_{i}$	21.1273	19.9715	22.3182
	$p_{i}$	0.4	0.56	0.58		$p_{i}$	0.6	0.54	0.52
8	$L_{i}$	32.9091	21.7139	52.7546	8	$L_{i}$	42.1394	35.9509	44.1636
	$p_{i}$	0.6	0.5	0.54		$p_{i}$	0.6	0.5	0.54
9	$L_{i}$	16.9151	14.2752	38.4412	9	$L_{i}$	24.4364	21.9733	27.5012
	$p_{i}$	0.6	0.52	0.54		$p_{i}$	0.4	0.54	0.54
10	$L_{i}$	30.0727	14.7309	21.1418	10	$L_{i}$	16.3152	18.5473	19.3721
	$p_{i}$	0.2	0.52	0.52		$p_{i}$	0.6	0.54	0.5
11	$L_{i}$	29.3212	18.7345	50.537	11	$L_{i}$	36.4909	36.4655	42.06
	$p_{i}$	0.6	0.64	0.44		$p_{i}$	0.6	0.5	0.42
12	$L_{i}$	21.6424	13.2697	34.1182	12	$L_{i}$	17.9817	19.143	20.5849
	$p_{i}$	0.6	0.62	0.52		$p_{i}$	0.4	0.6	0.54
13	$L_{i}$	39.8727	19.3085	31.3618	13	$L_{i}$	30.406	28.7436	16.9236
	$p_{i}$	0.6	0.6	0.48		$p_{i}$	0.6	0.52	0.52
14	$L_{i}$	25.503	23.3364	50.7218	14	$L_{i}$	24.9576	36.1054	39.3079
	$p_{i}$	0.6	0.56	0.48		$p_{i}$	0.6	0.46	0.58
15	$L_{i}$	19.0424	22.0957	54.723	15	$L_{i}$	37.6788	33.1055	37.7097
	$p_{i}$	0.4	0.48	0.36		$p_{i}$	0.4	0.44	0.5

**Table 8:** The information entropy $H1_{i}$ and $H2_{i}$ of 15 recognition parameters of 500 training images.

The Number of Training Iris Is 500					The Number of Training Iris Is 100				
No.		JLU-4.0	JLU-6.0	JLU-7.0	No.		JLU-4.0	JLU-6.0	JLU-7.0
1	$H1_{i}$	0.306495	0.315942	0.315942	1	$H1_{i}$	0.306495	0.352305	0.324698
	$H2_{i}$	0.366516	0.36435	0.36435		$H2_{i}$	0.366516	0.340042	0.361231
2	$H1_{i}$	0.366516	0.352305	0.352305	2	$H1_{i}$	0.366516	0.352305	0.346574
	$H2_{i}$	0.306495	0.340042	0.340042		$H2_{i}$	0.306495	0.340042	0.346574
3	$H1_{i}$	0.366516	0.315942	0.346574	3	$H1_{i}$	0.306495	0.315942	0.340042
	$H2_{i}$	0.306495	0.36435	0.346574		$H2_{i}$	0.366516	0.36435	0.352305
4	$H1_{i}$	0.306495	0.332741	0.352305	4	$H1_{i}$	0.306495	0.332741	0.352305
	$H2_{i}$	0.366516	0.357203	0.340042		$H2_{i}$	0.366516	0.357203	0.340042
5	$H1_{i}$	0.366516	0.352305	0.36435	5	$H1_{i}$	0.366516	0.324698	0.352305
	$H2_{i}$	0.306495	0.340042	0.315942		$H2_{i}$	0.306495	0.361231	0.340042
6	$H1_{i}$	0.366516	0.315942	0.357203	6	$H1_{i}$	0.366516	0.357203	0.306495
	$H2_{i}$	0.306495	0.36435	0.332741		$H2_{i}$	0.306495	0.332741	0.366516
7	$H1_{i}$	0.366516	0.324698	0.315942	7	$H1_{i}$	0.306495	0.332741	0.340042
	$H2_{i}$	0.306495	0.361231	0.36435		$H2_{i}$	0.366516	0.357203	0.352305
8	$H1_{i}$	0.306495	0.346574	0.332741	8	$H1_{i}$	0.306495	0.346574	0.332741
	$H2_{i}$	0.366516	0.346574	0.357203		$H2_{i}$	0.366516	0.346574	0.357203
9	$H1_{i}$	0.306495	0.340042	0.332741	9	$H1_{i}$	0.366516	0.332741	0.332741
	$H2_{i}$	0.366516	0.352305	0.357203		$H2_{i}$	0.306495	0.357203	0.357203
10	$H1_{i}$	0.321888	0.340042	0.340042	10	$H1_{i}$	0.306495	0.332741	0.346574
	$H2_{i}$	0.178515	0.352305	0.352305		$H2_{i}$	0.366516	0.357203	0.346574
11	$H1_{i}$	0.306495	0.285624	0.361231	11	$H1_{i}$	0.306495	0.346574	0.36435
	$H2_{i}$	0.366516	0.367794	0.324698		$H2_{i}$	0.366516	0.346574	0.315942
12	$H1_{i}$	0.306495	0.296382	0.340042	12	$H1_{i}$	0.366516	0.306495	0.332741
	$H2_{i}$	0.366516	0.367682	0.352305		$H2_{i}$	0.306495	0.366516	0.357203
13	$H1_{i}$	0.306495	0.306495	0.352305	13	$H1_{i}$	0.306495	0.340042	0.340042
	$H2_{i}$	0.366516	0.366516	0.340042		$H2_{i}$	0.366516	0.352305	0.352305
14	$H1_{i}$	0.306495	0.324698	0.352305	14	$H1_{i}$	0.306495	0.357203	0.315942
	$H2_{i}$	0.366516	0.361231	0.340042		$H2_{i}$	0.366516	0.332741	0.36435
15	$H1_{i}$	0.366516	0.352305	0.367794	15	$H1_{i}$	0.366516	0.361231	0.346574
	$H2_{i}$	0.306495	0.340042	0.285624		$H2_{i}$	0.306495	0.324698	0.346574

**Table 9:** The 15 values of the category labels of the three iris libraries.

The Number of Training Iris Is 500				The Number of Training Iris Is 100			
No.	JLU-4.0	JLU-6.0	JLU-7.0	No.	JLU-4.0	JLU-6.0	JLU-7.0
1	0.330504	0.336273	0.336273	1	0.330504	0.345928	0.340773
2	0.330504	0.345928	0.345928	2	0.330504	0.345928	0.346574
3	0.330504	0.336273	0.346574	3	0.330504	0.336273	0.345928
4	0.330504	0.343993	0.345928	4	0.330504	0.343993	0.345928
5	0.330504	0.345928	0.336273	5	0.330504	0.340773	0.345928
6	0.330504	0.336273	0.343993	6	0.330504	0.343993	0.330504
7	0.330504	0.340773	0.336273	7	0.330504	0.343993	0.345928
8	0.330504	0.346574	0.343993	8	0.330504	0.346574	0.343993
9	0.330504	0.345928	0.343993	9	0.330504	0.343993	0.343993
10	0.207189	0.345928	0.345928	10	0.330504	0.343993	0.346574
11	0.330504	0.315205	0.340773	11	0.330504	0.346574	0.336273
12	0.330504	0.323476	0.345928	12	0.330504	0.330504	0.343993
13	0.330504	0.330504	0.345928	13	0.330504	0.345928	0.345928
14	0.330504	0.340773	0.345928	14	0.330504	0.343993	0.336273
15	0.330504	0.345928	0.315205	15	0.330504	0.340773	0.346574

**Table 10:** The recognition parameters of the training iris of the example.

No.	JLU-4.0	JLU-6.0	JLU-7.0	No.	JLU-4.0	JLU-6.0	JLU-7.0
1	35.7268	12.788	12.9091	9	21.8484	24.0909	19.1818
2	30.7579	24.5452	55.6973	10	8.36367	22.2121	7.5758
3	24.9394	22.3033	26.9697	11	22.3636	24.3636	47.606
4	28.3939	13.1515	15.1817	12	15.2727	7.9696	23.2121
5	28.0303	25.606	56.2121	13	15.7272	4.06066	10.4243
6	21.7576	21.9091	51.4849	14	23.3637	13.6667	40.4849
7	19.8787	11.2424	26.9091	15	5.93933	11.1515	56.1819
8	22.8181	6.99993	48.4243				

**Table 11:** The information offset $Z_{i}$ of the example training iris.

The Number of Training Iris Is 500				The Number of Training Iris Is 100			
No.	JLU-4.0	JLU-6.0	JLU-7.0	No.	JLU-4.0	JLU-6.0	JLU-7.0
1	0.15695	0.149091	0.0970519	1	0.399367	0.114928	0.0917578
2	0.117914	0.165834	0.139936	2	0.0796696	0.0911246	0.178281
3	0.303185	0.203128	0.0944764	3	0.168877	0.147454	0.134772
4	0.0957285	0.122894	0.078089	4	0.152059	0.0874362	0.158619
5	0.201546	0.187428	0.18785	5	0.113255	0.114782	0.209799
6	0.122257	0.160448	0.153639	6	0.0791217	0.106853	0.179213
7	0.0750067	0.146459	0.158722	7	0.173029	0.101146	0.203892
8	0.127508	0.0558626	0.164931	8	0.0995787	0.0337403	0.180166
9	0.189364	0.285385	0.0896586	9	0.13108	0.180149	0.125325
10	0.0179044	0.254989	0.063361	10	0.0942713	0.196781	0.0677668
11	0.14026	0.17219	0.149724	11	0.112702	0.115778	0.207409
12	0.129773	0.110362	0.1203	12	0.12452	0.07656	0.185284
13	0.0725356	0.0386744	0.056209	13	0.095119	0.0249799	0.108916
14	0.168471	0.106487	0.134977	14	0.172153	0.0621963	0.157609
15	0.0457266	0.0853465	0.187673	15	0.0231097	0.0535391	0.258172

**Table 12:** The entropy feature G and enlarging value $e^{G}$.

The Number of Training Iris Is 500				The Number of Training Iris Is 100			
No.	JLU-4.0	JLU-6.0	JLU-7.0	No.	JLU-4.0	JLU-6.0	JLU-7.0
G	11.9502	13.2395	11.0544	G	12.2111	8.7975	14.2748
$e^{G}$	154,844	562,137	63,220.3	$e^{G}$	201,013	6617.71	1,583,010

**Table 13:** The results of the quality evaluation and recognition of the four cases.

Case		JLU-4.0	JLU-6.0	JLU-7.0
1	Qualified quantity	937	953	964
	Accuracy	42.39%	47.53%	28.75%
2	Qualified quantity	975	946	968
	Accuracy	44.53%	42.85%	32.56%
3	Qualified quantity	986	986	972
	Accuracy	37.59%	39.42%	41.25%
4	Qualified quantity	903	863	893
	Accuracy	50.67%	58.25%	49.53%

**Table 14:** Feedback and updates.

No.	Training Iris Number	Test Iris Number	Number of Correct Certification	Correct Certification Rate	Whether to Update Judgment
JLU-4.0					
1	10	50	48	96%	No
2	10	100	69	69%	Yes
3	100	200	189	94.5%	Yes
4	500	200	200	100%	No
JLU-6.0					
1	10	50	38	76%	Yes
2	100	200	193	96.5%	No
3	100	500	375	75%	Yes
4	500	500	499	99.8%	No
JLU-7.0					
1	10	50	42	84%	Yes
2	50	100	89	89%	Yes
3	200	200	198	99%	No
4	200	500	423	84.6%	Yes

**Table 15:** The change situation of category label range distribution.

The Number of Training Iris	Category Label Range
**JLU-4.0**	
10	[1000,2000]
100	[1000,2000],[1800,1900]
500	[1000,2000],[1800,1900],[13000,14000],[8900,9000]
JLU-6.0	
10	[890,000,900,000],[310,000,320,000],
50	[890,000,900,000],[310,000,320,000],[138,000,000,139,000,000],[1,820,000,1,830,000]
100	[890,000,900,000],[310,000,320,000],[138,000,000,139,000,000],[1,820,000,1,830,000], [2,000,000,2,100,000]
500	[890,000,900,000],[310,000,320,000],[138,000,000,139,000,000],[1,820,000,1,830,000], [2,000,000,2,100,000],[1,190,000,1,200,000],[125,000,000,126,000,000]
JLU-7.0	
10	[138,000,139,000],[1,150,000,1,160,000]
50	[138,000,139,000],[1,150,000,1,160,000],[3,500,000,3,800,000]
100	[138,000,139,000],[1,150,000,1,160,000],[3,500,000,3,800,000],[2,900,000,3,000,000], [840,000,850,000]
200	[138,000,139,000],[1,150,000,1,160,000],[3,500,000,3,800,000],[2,900,000,3,000,000], [840,000,850,000],[2,470,000,2,480,000],[720,000,730,000]
500	[138,000,139,000],[1,150,000,1,160,000],[3,500,000,3,800,000],[2,900,000,3,000,000], [840,000,850,000],[2,470,000,2,480,000],[720,000,730,000]

**Table 16:** The results of the heterogeneous universality experiment.

		Total Number of Collection	Quality Error	False Rejection	False Acceptance
JLU-4.0	Category 1	105	3	2	0
	Category 2	107	5	1	1
	Category 3	103	0	3	0
	Category 4	101	1	0	0
	Category 5	106	2	3	1
JLU-6.0	Category 1	101	0	1	0
	Category 2	100	0	0	0
	Category 3	103	1	1	1
	Category 4	100	0	0	0
	Category 5	104	1	3	0
JLU-7.0	Category 1	105	1	3	1
	Category 2	109	5	4	0
	Category 3	104	1	2	1
	Category 4	107	0	7	0
	Category 5	105	2	3	0

**Table 17:** Certification times of each category in different iris libraries.

		The Same Category	Different Category
**JLU-4.0**	Category 1	1756	8435
	Category 2	1657	9135
	Category 3	1568	9253
	Category 4	1456	8965
	Category 5	1536	9568
JLU-6.0	Category 1	1478	8965
	Category 2	1569	8567
	Category 3	1745	9542
	Category 4	1869	9645
	Category 5	1756	9456
JLU-7.0	Category 1	1689	7895
	Category 2	1756	8456
	Category 3	1896	9674
	Category 4	1598	8456
	Category 5	1796	8695

**Table 18:** The results of the time operation experiment.

Iris Library	Algorithm Running Time in This Paper	Recognizable Irises Considered by Evaluation	The Correct Number of Recognizable	Unrecognizable Irises Considered by Evaluation	The Correct Number of Unrecognizable
JLU-4.0	6186 ms	786	785	214	0
JLU-6.0	6347 ms	815	815	185	0
JLU-7.0	6412 ms	868	868	132	0

**Table 19:** The number of iris categories and the number of single-category irises.

Category Number	Image Number in Each Category	Total	Match Number in the Same Category	Match Number in Different Category	Total
50	100	5000	5000	10,000	15,000

**Table 20:** The number of qualified irises in the three iris libraries developed in three different ways.

	Recognizable Iris Considered by Evaluation	Certified Ratio	Unrecognizable Iris Considered by Evaluation
JLU-4.0			
Qualified iris evaluation indicators in literature [41]	9135	60.9%	5865
Inference engine system in literature [40]	9635	64.233%	5365
Quality evaluation fuzzy reasoning system(example indicators in this paper)	13,585	90.567%	1415
JLU-6.0			
Qualified iris evaluation indicators in literature [41]	8123	54.153%	6877
Inference engine system in literature [40]	7658	51.053%	7342
Quality evaluation fuzzy reasoning system(example indicators in this paper)	14,325	95.5%	675
JLU-7.0			
Qualified iris evaluation indicators in literature [41]	6635	44.233%	8365
Inference engine system in literature [40]	7468	49.787%	7532
Quality evaluation fuzzy reasoning system(example indicators in this paper)	12,453	83.02%	2547

**Table 21:** The recognition results of all 12 cases in the three libraries.

Case	The Correct Number of Recognizable Irises	CRR of Recognizable	The Correct Number of Unrecognizable Irises	CRR of Unrecognizable
JLU-4.0				
0	13,585	100%	1	0.0707%
1	6842	74.899%	1125	19.182%
2	7368	80.657%	426	7.263%
3	8568	88.928%	536	9.991%
4	8356	91.472%	1365	23.274%
5	6789	70.462%	145	2.703%
6	8756	90.877%	3658	68.183%
7	10,685	78.653%	85	6.007%
8	9756	71.815%	12	0.848%
9	11,423	84.085%	6	0.424%
10	13,086	96.327%	10	0.707%
11	10,985	80.861%	9	0.636%
12	10,586	77.924%	23	1.625%
JLU-6.0				
0	14,323	99.986%	0	0%
1	6135	75.526%	1574	22.888%
2	6585	81.066%	589	8.565%
3	6987	91.237%	458	6.238%
4	7162	88.169%	2869	41.719%
5	5869	76.639%	95	1.294%
6	7436	97.101%	3658	49.823%
7	11,468	80.056%	25	3.704%
8	10,125	70.681%	3	0.444%
9	11,987	83.678%	5	0.741%
10	13,845	96.649%	0	0%
11	12,452	86.925%	0	0%
12	12,653	88.328%	5	0.741%
JLU-7.0				
0	12,452	99.992%	2	0.079%
1	4785	72.118%	1023	12.230%
2	4985	75.132%	523	6.252%
3	6723	90.024%	354	4.700%
4	5621	84.717%	1862	22.259%
5	5862	78.495%	115	1.527%
6	7256	97.161%	3675	48.792%
7	9926	79.708%	1	0.039%
8	9352	75.098%	3	0.118%
9	9789	78.607%	1	0.039%
10	11,589	93.062%	1	0.039%
11	10,985	88.212%	3	0.118%
12	10,709	85.995%	4	0.157%
